# Amelioration of experimental autoimmune encephalomyelitis through transplantation of placental derived mesenchymal stem cells

**DOI:** 10.1038/srep41837

**Published:** 2017-02-10

**Authors:** Hong Jiang, Yuanyuan Zhang, Kewei Tian, Beibei Wang, Shu Han

**Affiliations:** 1 Department of Electrophysiology, Sir Run Run Shaw Hospital, Medical College, Zhejiang University, Hangzhou, China; 2 Institute of Anatomy and Cell Biology, Medical College, Zhejiang University, Hangzhou, China; 3 Core Facilities, Zhejiang University School of Medicine, Hangzhou, China

## Abstract

Placental derived mesenchymal stem cells (PMSCs) have been suggested as a possible source of cells to treat multiple sclerosis (MS) due to their immunomodulatory functions, lack of ethical concerns, and potential to differentiate into neurons and oligodendrocytes. To investigate whether PMSCs share similar characteristics with embryonic mesenchymal stem cells (EMSCs), and if transplanted PMSCs have the ability to integrate and replace degenerated neural cells, we transplanted rat PMSCs and EMSCs into the central nervous system (CNS) of Lewis rats with experimental autoimmune encephalomyelitis (EAE), an animal model of MS. Our findings demonstrated that transplanted PMSCs, similar to EMSCs, were effective in decreasing infiltrating inflammatory cells, preserving axons, and ameliorating demyelination, thereby improving the neurological functions of animals. Moreover, both PMSCs and EMSCs had the ability to migrate into inflamed tissues and express neural–glial lineage markers. These findings suggest that PMSCs may replace EMSCs as a source of cells in MS stem cell therapy.

Multiple sclerosis (MS) is an autoimmune disease characterized by aberrant activation of immune cells, which causes demyelination, axonal damage, and inflammation in the central nervous system (CNS)^1^,^2^,^3^. MS most often affects young females and causes a variety of neurological disabilities with a relapsing-remitting course. To date, treatments target symptoms^4^, rather than providing curative options^5^.

Recently, clinical trials in MS patients have evaluated the therapeutic potential of mesenchymal stem cells (MSCs) derived from a variety of sources, such as bone marrow (BM), adipose tissue, placenta and umbilical cord blood^6^,^7^. Some studies have shown structural, functional, and physiological improvements after treatment, and these improvements are attributed to the immunomodulatory and neuroprotective effects of MSCs^8^,^9^. Compared with MSCs from adult donors, MSCs from less developmentally advanced sources have a higher potential to proliferate and a greater propensity to differentiate. MSCs can, therefore, serve as an unlimited source of neural cells for transplantation in neurological disorders^10^,^11^. MSCs from more developmentally naïve cells, such as embryonic mesenchymal stem cells (EMSCs), could obviate the need for constant donor recruitment, and reduce the risk of complications associated with multiple donors^12^,^13^. However, ethical conflicts associated with the use of EMSCs have limited their application. In the last decade, decidua-derived MSCs (DMSCs)^14^ and placental derived mesenchymal stem cells (PMSCs) have been considered as ideal sources for MSCs^15^. Although PMSCs have shown therapeutic effects in an animal model of MS^15^, the underlying mechanisms by which they exert their action are still unknown.

The acute experimental allergic encephalomyelitis (EAE) model induced in Lewis rats is a well-established model of MS, and is characterized by a single peak of paralysis after which animals recover spontaneously^6^. Thus, this model provides a more convenient way to mimic the entire process of induction, peak, and resolution of the inflammatory response associated with MS than the classical mouse model by MOG35–55 induction, in which Selim and colleagues have tested and provided some evidence of neuroprotective effects with full-term human placenta (PDMSCs)^16^.

To compare the efficiency of EMSCs and PMSCs in treating MS and to test the integrative capacity of transplanted EMSCs and PMSCs, in the present study, we transplanted PMSCs from green fluorescent protein (GFP) transgenic rats into the CNS of EAE rats through bilateral intracerebroventricular (ICV) injections and intrathecal (ITH) injection. EMSCs, which have been previously demonstrated to have some therapeutic efficacy in the EAE model, were used as the positive control^12^,^17^,^18^. Multiple behavioral and neurological evaluations, histological and immunohistochemical staining, enzyme-linked immunosorbent assays (ELISA), Western blotting, electron microscopy (EM), and electrophysiological tests were adopted to assess a variety of parameters, including inflammation, axonal loss, white matter demyelination, neuronal apoptosis, gliosis, expression of pro-inflammatory cytokines, functional recovery of treated EAE rats, as well as the survival, migration, and differentiation of engrafted PESCs and EMSCs in the cerebral cortex and spinal cord of EAE rats.

## Results

### Differentiation potential of PMSCs

PMSCs have the potential to differentiate into all cell types, depending on the local microenvironment^15^. To test the ability of our PMSCs to differentiate into neural cells before the transplantation, we cultured cells in the neural differentiation medium, and stained the cells with specific neural markers. As expected, our cultured PMSCs extensively co-expressed the mesenchymal stem cell marker CD44 (red) along with the astrocyte specific marker GFAP (green, Figure S1A–D), oligodendrocyte specific marker Olig1 (green, Figure S1E–H), or neuron specific marker NF-200 (green, Figure S1M–Q). Partial expression of the microglia/macrophage specific marker CD68 (green, Figure S1I–L) was also present. The results suggest that our PMSCs have the potential to differentiate into both neuronal and glia cells *in vitro*.

### Both EMSCs and PMECs treatments reverse electrophysiological dysfunction, postpone the onset of motor symptoms, and reduce disease severity in EAE rats

**Figure 1:**
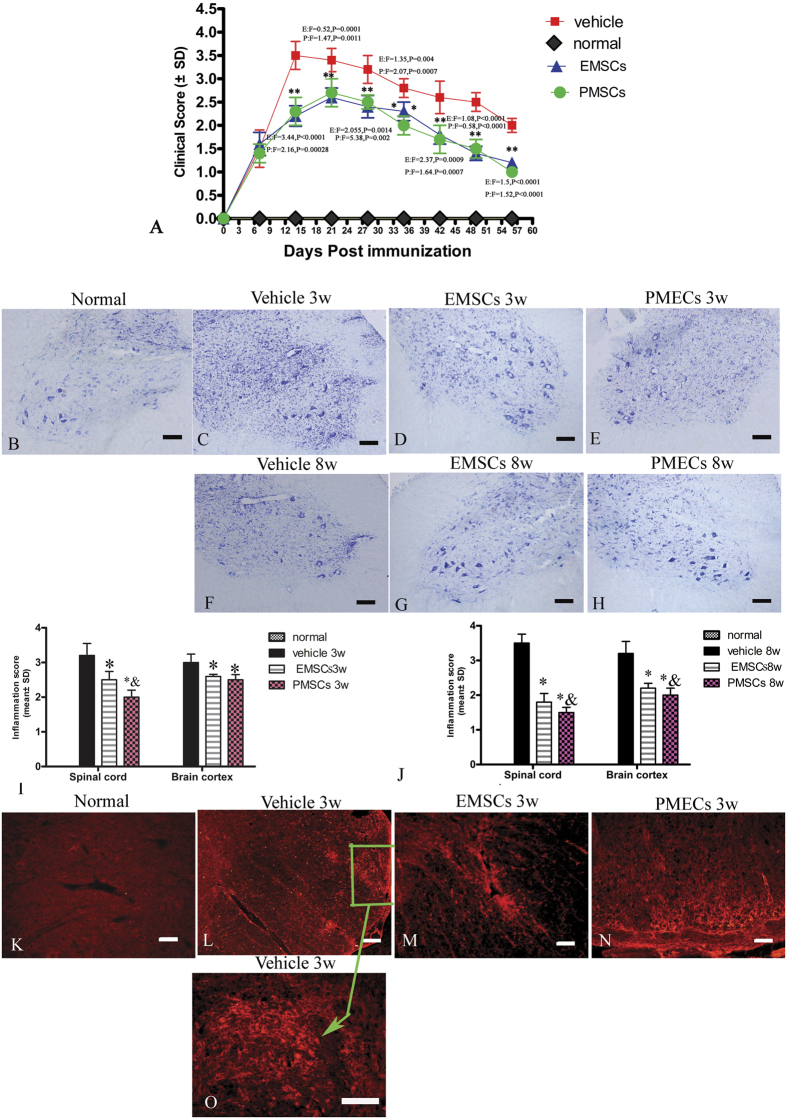
EMSCs and PMSCs treatments inhibit inflammatory cell infiltration and delay clinical progression of EAE. Ten days after EAE induction, rats received bilateral ICV injections of 1 × 10^6^ PMSCs, EMSC or phosphate-buffered saline (PBS) at AP + 0.6 mm, ML ± 0.7 mm, and V −3 mm, from Bregma based on the mouse stereotaxic atlas (Paxinos and Watson). For ITH transplantation, EMSCs, PMECs (1 × 10^6^ cells) or saline were injected intrathecally in the lumbar spinal cord (L4–L5). The clinical and inflammation scoring were repeated three times. (**A**) Both EMSCs and PMECs treatment postpone the onset of motor symptoms and reduce disease severity in EAE rats as measured by disease scoring. Data are represented as mean ± SD. n = 5, degrees of freedom = 4 E: EMSCs transplanted group; P: PMECs transplanted group. (**B–H**) Nissel staining showed diffuse infiltration of inflammatory cells in the spinal cord of the vehicle treated EAE rats, which was attenuated in EMSCs and PMSCs transplanted rats. (**K–O**) infiltration of inflammatory CD68+ (a marker for extravasated microcytes/macrophages) cells were observed surrounding blood vessels and in the parenchyma of spinal cord (green arrow in O) in vehicle-treated EAE rats. Both the EMSCs and PMSCs treatments could alleviate the infiltration. CD68 TRIFC-immunofluorescence staining (red). Scale bar = 100 μm. (**I,J**) EMSCs and PMSCs treatments attenuated CNS inflammation at 3 (I) and 8 (J) weeks post-injection, as shown by inflammation scoring. Data are represented as mean ± SD. n = 5, degrees of freedom = 4. *P < 0.01 *Vs* vehicle group (3w PI, in SP, EMSCs group: F = 1.23, P = 0.0004; PMECs group: F = 2.37, P < 0.0001. In BC, EMSCs group: F = 0.44, P = 0.00015; PMECs group: F = 0.068, P = 0.0033; 8 W PI, in SP, EMSCs group: F = 3.3, P < 0.0001; PMECs group: F = 0.18, P < 0.0001. In BC, EMSCs group: F = 3.46, P < 0.0001; PMECs group: F = 0.83, P < 0.0001). ^&^P < 0.05 *Vs* EMSCs group (3 W PI, In SP, PMECs group: F = 0.52, P = 0.0086; 8 W PI, In SP, PMECs group: F = 0.61, P = 0.00025).

To test the effects of EMSCs and PMSCs in treating neurological dysfunction in EAE rats, we assessed rats with a functional scoring after cellular transplantation. The functional scoring results demonstrated that, in vehicle-treated rats, disease symptoms developed 9–10 days after injection (>2.0), and the acute phase began with a sharp increase in the severity of motor symptoms (average clinical score of 3.5–4.0), which peaked at 2 weeks post-injection. Thereafter, clinical scores gradually declined and acute EAE rats underwent spontaneous recovery. Eight weeks after injection, the clinical scores of the vehicle-treated animals returned to the level of 2. In the EMSCs and PMECs transplant group, disease symptoms also appeared on 9–10 days post- injection, consistent with the vehicle treated group. However, 10 days post-injection, disease progression in the EMSCs and PMECs-treated groups showed a reduced disease-slope and the peak stage of the disease was postponed to 3 weeks after the injection. The clinical scores at each time point were markedly lower in these two groups than in the vehicle-treated controls from 3 weeks to the spontaneous recovery stage (Fig. 1A).

Somatosensory evoked potential (SEP) and motor evoked potentials (MEP) have been used to evaluate neural damage in MS patients^19^,^20^,^21^. To test the effects of EMSCs and PMSCs transplantation on sensory and motor functions in EAE rats, we recorded the SEP and MEP after transplantation. EAE induction prolonged the latency to waveform initiation and decreased peak amplitude in both cortical somatosensory evoked potential (c-SEP; Table 1) and MEP (Table 1) recordings. However, transplantation of both EMSCs and PMSCs significantly attenuated the severity of electrophysiological disturbances by reducing disease-associated delays in latency related to the speed of conduction and reversing the decrease in amplitude related to the number of surviving fibers (Table 1, Figure S2).

### EMSCs and PMECs treatment attenuates perivascular/parenchymal infiltration and reduces CNS inflammation

**Figure 2:**
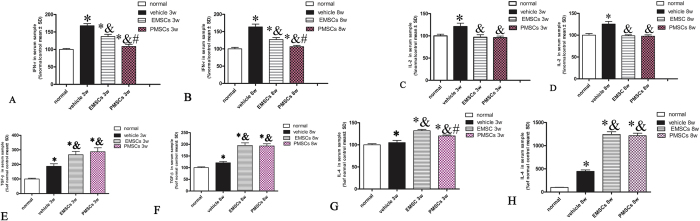
EMSCs and PMECs treatment suppress pro-inflammatory factors and transcription factors in inflammatory pathway, but increase the expression of anti-inflammatory cytokines. EMSCs and PMSCs treatments effectively reduce the expression of pro-inflammatory factors IFN-γ *P < 0.01*Vs* Normal group (3w Vehicle group: F = 3.41, P < 0.0001;EMSCs group: F = 6.24, P < 0.0001, PMECs group: F = 0.18, P = 0.0053; 8w PI, Vehicle group: F = 0.23, P < 0.0001;EMSCs group: F = 0.74, P < 0.0001; PMECs group: F = 0.73, P = 0.0031) & P < 0.01 *Vs* Vehicle group (3w PI, EMSCs group: F = 0.54, P < 0.0001; PMECs group: F = 0.62, P < 0.0001. 8w PI, EMSCs group: F = 3.12, P < 0.0001; PMECs group: F = 0.13, P < 0.0001). ^#^P < 0.01 *Vs* EMSCs group (3w PI, PMSCs group: F = 1.14, P < 0.0001; 8w PI, PMSCs group: F = 0.43, P < 0.0001) (**A,B**) and IL-2 *P < 0.01*Vs* Normal group (3w Vehicle group: F = 0.22, P < 0.0001; 8w PI, Vehicle group: F = 0.6, P < 0.0001) & P < 0.01 *Vs* Vehicle group (3w PI, EMSCs group: F = 2.51, P < 0.0001; PMECs group: F = 0.8, P < 0.0001. 8w PI, EMSCs group: F = 0.4, P < 0.0001; PMECs group: F = 0.45, P < 0.0001) (**C,D**), but up regulated the expression of anti-inflammatory cytokines TGF-β *P < 0.01*Vs* Normal group (3w Vehicle group: F = 0.1, P < 0.0001;EMSCs group: F = 0.06, P < 0.0001. PMECs group: F = 0.043, P < 0.0001; 8w PI, Vehicle group: F = 0.39, P < 0.0001.EMSCs group: F = 0.11, P < 0.0001. PMECs group: F = 0.15, P < 0.0001).& P < 0.01 *Vs* Vehicle group (3w PI, EMSCs group: F = 0.58, P = 0.00013; PMECs group: F = 0.42, P < 0.0001. 8w PI, EMSCs group: F = 0.28, P < 0.0001; PMECs group: F = 0.4, P < 0.0001). (**E,F**) and IL-4 *P < 0.01*Vs* Normal group (3w Vehicle group: F = 0.14, P = 0.017; EMSCs group: F = 0.39, P < 0.0001, PMECs group: F = 0.46, P < 0.0001; 8w PI, Vehicle group: F = 0.0038, P < 0.0001; EMSCs group: F = 0.0017, P < 0.0001; PMECs group: F = 0.00167, P < 0.0001). ^&^P < 0.01 *Vs* Vehicle group (3w PI, EMSCs group: F = 1.25, P < 0.0001; PMECs group: F = 1.45, P < 0.0001. 8w PI, EMSCs group: F = 0.45, P < 0.0001; PMECs group: F = 0.44, P < 0.0001). ^#^P < 0.01 *Vs* EMSCs group (3w PI, PMSCs group: F = 0.86, P = 0.00017). Data are represented as mean ± SD. n = 5, degrees of freedom = 4.

Neural inflammation is a cardinal sign in both MS and EAE. To assess neural inflammation changes after EMSCs and PMSCs transplantation, we examined perivascular/paraenchymal infiltration of inflammatory cells by Cresyl violet and CD68 staining, and evaluated inflammation present by using a scoring system. At the peak stage of acute EAE (3 weeks post-injection), vehicle-treated rats exhibited a significant increase in infiltrating inflammatory cells. Diffuse infiltration of inflammatory cells appeared around blood vessels throughout the brain and spinal cord parenchyma and under the meninges (Fig. 1C). Reduced perivascular and parenchymal inflammatory infiltrate was observed in EMSCs-treated rats (Fig. 1D) and PMECs-treated rats (Fig. 1E). We also performed immunostaining of CD68, a marker for activated microglia and extravasated macrophages (Fig. 1K–O). A typical infiltration of macrophages in spinal cord parenchymal is shown in Fig. 2 (arrows in Fig. 2N). Similarly, EMSCs and PMECs treatment reduced the number of extravasated macrophages (Fig. 1M and N).

Eight weeks post-injection, the inflammatory cell infiltration in the vehicle-treated group decreased, as compared to those of the same group at 3 weeks after the injection (Fig. 1F). The number of extravasated inflammatory cells in EMSCs and PMECs-treated groups remained lower than those in the vehicle-treated group (Fig. 1G and H). The inflammatory scores of EMSCs and PMECs-treated groups were also significantly lower than that of the vehicle-treated EAE group at both 3 and 8 weeks after the injection (Fig. 1I and J).

### EMSCs and PMECs treatment suppress pro-inflammatory factors and transcription factors involved in inflammatory pathways and increase the expression of anti-inflammatory cytokines

The beneficial effects of EMSCs and PMSCs may be attributed to their anti-inflammatory functions following transplantation. Therefore, we examined the expression of pro-inflammatory and anti-inflammatory factors in inflammatory pathways. Expression levels of TGF-β, IFN-γ, IL-2, and IL-4 levels in blood serum were measured by enzyme-linked immunosorbent assay (ELISA Fig. 2) and the levels of COX-2, NF-kB, and TNF-α were detected by Western blotting and immunostaining (Figure S3) at the early (week 3) and late (week 8) stages of EAE. We found that expression of transcription factors in inflammatory pathways, such as COX-2 and NF-kB, and inflammatory cytokines, such as TNF-α, IFN-γ and IL-2, were all significantly increased in vehicle-treated EAE rats, while EMSCs and PMECs treatments markedly reduced the expression of these factors (Figures S3 and S4).

In contrast, the expression of the anti-inflammatory cytokines IL-4 and TGF-β were clearly down regulated in vehicle-treated EAE rats as compared with normal control rats at 3 and 8 weeks post-injection; EMSCs and PMECs treatments also reversed these effects (Fig. 2E–H).

### EMSCs and PMECs treatments inhibit demyelination, alleviate perivascular edema/leakage, and reduce neuronal necrosis and apoptosis

**Figure 3:**
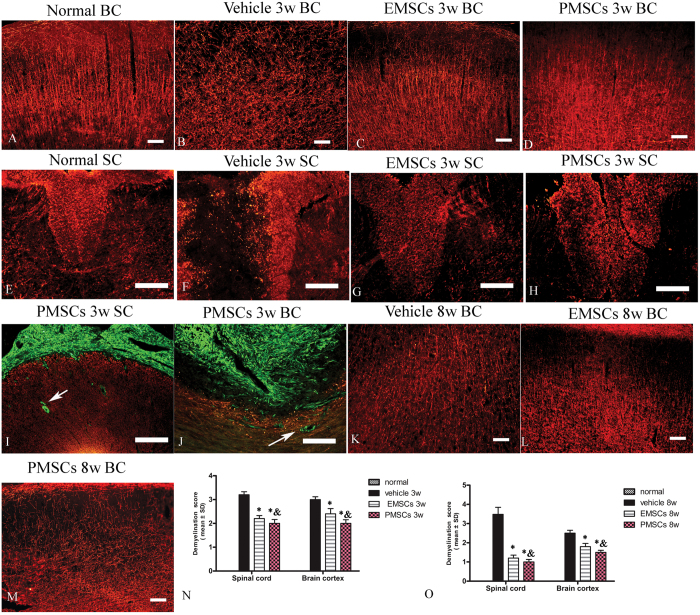
EMSCs and PMECs treatments inhibit demyelination. (**A–J**) At 3 weeks post-injection, myelin disruption, disorder and demyelination signs were revealed in the brain cortex and posterior funiculus of spinal cord in vehicle group (showed by MBP immunofluorescence, red), while EMSCs and PMSCs treatments reduced demyelination. In this stage, the transplanted EMSCs and PMSCs (GFP-conjugated, green) in subarachnoid space (**I**) and lateral ventricles (**J**) begin to infiltrate into spinal cord and brain tissue parenchymal (showed by arrows). (**K–M**) At 8 weeks post-injection, the vehicle treated group exhibited more pronounced demyelination and EMSCs and PMSCs treatments obviously reversed this phenomenon. Scale bar = 100 μm. (SC) Transverse sections through the anterior horn of the lumbar spinal. (BC) Coronal sections of the brain cortex. (**N,O**) The effect of EMSCs and PMSCs treatments on myelination at 3 weeks (**N**) and 8 weeks (**O**) post-immunization, as estimated by demyelination score. Data are represented as mean ± SD. n = 5, degrees of freedom = 4. *P < 0.05 *Vs* vehicle group (3w PI, in SP, EMSCs group: F = 1, P < 0.0001; PMECs group: F = 0.52, P < 0.0001. In BC, EMSCs group: F = 0.28, P = 0.0003; PMECs group: F = 0.63, P < 0.0001. 8 W PI, in SP, EMSCs group: F = 5.33, P P < 0.0001; PMECs group: F = 0.14, P < 0.0001. In BC, EMSCs group: F = 0.2, P < 0.0001; PMECs group: F = 0.55, P < 0.0001) & P < 0.05 *Vs* EMSCs group (3 W PI, In SP, PMECs group: F = 0.65, P = 0.03. In BC, PMECs group: F = 0.24, P = 0.0044. 8 W PI, In SP, PMECs group: F = 1.39, P = 0.04. In BC, PMECs group: F = 2.18, P = 0.005).

Demyelination, perivascular edema, and neural apoptosis are pathological characteristics of EAE rats at the cellular level. To test whether EMSCs and PMSCs transplantation ameliorated these pathological changes in EAE rats, we examined the morphological changes of the myelin sheath, blood vessels, and neurons by myelin basic protein (MBP) staining, and transmission electron microscopy. Western blotting and immunohistochemical labeling for MBP, a marker of myelination (Figs 2R–T and 3), was used to assess myelination in each group. An evident decrease in MBP expression and myelin disruption, disorder and demyelination were found in the cortex and posterior funiculus of the spinal cord in EAE rats, compared to control rats, both at 3 and 8 weeks post-injection (Figs 2R-T and B, F and K). However, EMSCs and PMSCs treatments resulted in a visible larger myelinated area and markedly reduced demyelination scores (Fig. 3C,D,G,H,L,M,N and O) and, furthermore, reduced the loss of MBP expression as shown by Western blotting (Fig. 2R and S) at both 3 and 8 weeks after the injection.

At 3 weeks post-injection, we demonstrated that transplanted EMSCs and PMSCs (GFP-conjugated, green) in the subarachnoid space (Fig. 3I) and lateral ventricles (Fig. 3J) had begun to infiltrate into the spinal cord and brain tissue parenchymal (shown by arrows), however, the expression of MBP in transplanted MSCs was difficult to detect.

**Figure 4:**
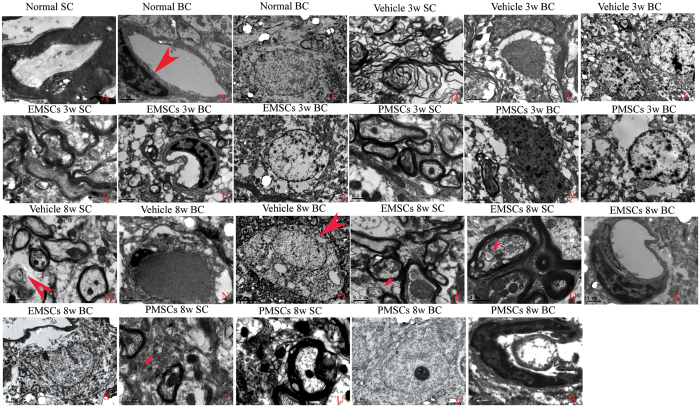
Electron micrograph demonstrating prevention of perivascular edema, demyelination/axon loss, and neuronal apoptosis in EMSCs and PMSCs-treated EAE rats. (**A–C**) Control rats. (**A**) Normal myelinated axons exhibited dark, ring-shaped myelin sheaths surrounding axons; (**B**) blood vessel with normal shapes, arrow indicates an EC; (**C**) normal neuronal nuclei with uncondensed chromatin; (**D–F**) vehicle-treated EAE rats 3 weeks postimmunization. (**D**) Myelin sheaths displayed splitting, vacuoles, loose and fused changes, and shrunken, atrophied axons; (**E**) Severe blood vessel leakage and tissue edema was detected in the extracellular space surrounding the vessels; (**F**) Neuron showing apoptotic signs with a shrunken nucleus and condensed, fragmented, and marginated nuclear chromatin. In EMSCs- (**G**–**I**), and PMSCs- (**J–L**) treated EAE rats 3 weeks post-injection, visible perivascular edema and leakage was still present (**H,K**), but the myelin sheaths splitting (**G,J**) and nucleus apoptotic signs (**I,L**) are evidently alleviated when compared with the vehicle treated EAE rats. At 8 weeks post-injection, demyelination and remyelination (arrow in M) appear simultaneously in vehicle-treated rats. Additionally, perivascular edema and leakage also was present in vehicle treated EAE rats (**N**). (**O**) A necrotic neuron with many large vacuoles and degenerated organelles in the perikaryon, rupturing cytoplasmic membrane, and oncolytic chromatin (arrow). In contrast, in EMSCs- (**P–S**) and PMSCs- (**T–W**), demyelination phenomenon is not evident and newly formed myelin sheaths surrounding intact axons (red arrows in **P,Q,T**), the morphology nucleus are relatively normal (**S,V**). The edema and leakage of blood vessels are also obviously ameliorated (**R,W**). (o) scale bar = 5 μm; (**C,E,F,I,K,L,M,S,V**), scale bar = 2 μm; (**A,B,D,H,N,Q,R,W**), scale bar = 1 μm; (**G,J,P,T,U**), scale bar = 0.5 μm. (SC) Transverse sections through the anterior horn of the lumbar spinal. (BC) Coronal sections of the brain cortex.

Transmission electron microscopy (TEM) demonstrated that in controls, myelin distribution, axon, and cell nuclei all exhibited normal cellular morphology (Fig. 4A and C), along with the absence of edema around blood vessels of control rats (Fig. 4B). However, in vehicle-treated EAE rats, myelin displayed significant splitting and vacuolar changes (Fig. 4D), and high levels of edema (Fig. 4E) were also detected in the extracellular space surrounding blood vessels. Furthermore, neurons demonstrated signs of apoptosis (Fig. 4F) at 3 weeks post-injection. In EMSCs and PMSCs-treated EAE rats, we also observed localized edema (Fig. 4H and K), however, splitting of the myelin sheath (Fig. 4G and J), as well as apoptotic signs (Fig. 4I and L), were evidently alleviated compared with vehicle treated EAE rats. At 8 weeks after the injection, demyelination and remyelination (arrow in Fig. 4M) appeared simultaneously in vehicle-treated rats. Additionally, perivascular edema and leakage were also present in vehicle treated EAE rats (Fig. 4N). A necrotic neuron is demonstrated in Fig. 4 with many large vacuoles and degenerated organelles in the perikaryon, ruptured cytoplasmic membrane, and oncolytic chromatin (Fig. 4O, arrows). On the contrary, in EMSCs- (Fig. 4P–S) and PMSCs-treated rats (Fig. 4T–W), demyelination was not evident and newly formed myelin sheaths surrounded intact axons (Red arrows in Fig. 4P,Q and T). Indeed, the morphology of the nuclei was abnormal (Fig. 4S and V) and the edema and leakage of blood vessels was also obviously ameliorated (Fig. 4R and W).

### EMSCs and PMECs treatments prevent axon loss

**Figure 5:**
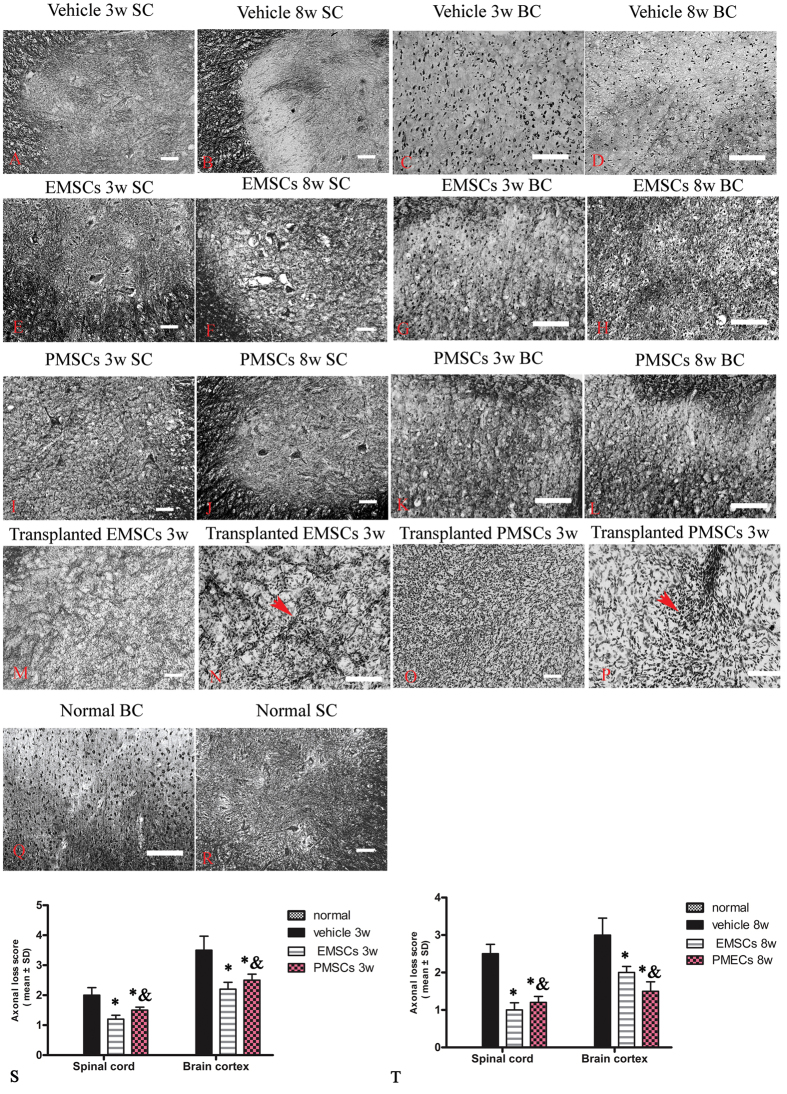
EMSCs and PMSCs treatments alleviate axonal loss at both 3 weeks and 8 weeks post-immunization demonstrated by Bielschowsky’s silver staining. (**A–D**) Numerous axons undergoing gradual loss both in brain cortex and spinal cord in vehicle-treated rats at 3 weeks and 8 weeks post-immunization. In EMSCs- (**E–H**) and PMSCs- (**I–L**) treated rats, more axons are retained relative to the vehicle-treated group at both 3 weeks (**S**) and 8 weeks (**T**) post-immunization, as estimated by axonal loss score. (**Q–R**) Normal control. Moreover, within the transplanted EMSCs (**M–N**) and PMSCs (**O–P**), the silver stained axons like fibers were found in both groups, as showed by arrows. Scale bar = 100 μm. Data are represented as mean ± SD. n = 5, degrees of freedom = 4. *P < 0.05 *Vs* vehicle group (3 W PI, in SP, EMSCs group: F = 3.56, P < 0.0001; PMECs group: F = 6.76, P = 0.003. In BC, EMSCs group: F = 4.23, P = 0.0003; PMECs group: F = 23.72, P = 0.003. 8 W PI, in SP, EMSCs group: F = 1.84, P < 0.0001; PMECs group: F = 2.34, P < 0.0001. In BC, EMSCs group: F = 7.63, P = 0.003; PMECs group: F = 3.35, P < 0.0001.) & P < 0.05 *Vs* EMSCs group (3 W PI, In SP, PMECs group: F = 1.9, P = 0.001. In BC, PMECs group: F = 0.8, P = 0.03. 8 W PI, in BC, PMECs group: F = 2.28, P = 0.002).

Axonal loss occurs in MS patients following myelin sheath loss. To examine the axonal changes after the EMSCs and PMSCs transplantation, we assessed the loss by Bielschowsky’s silver staining, a classical method to detect axonal degeneration. The results revealed reductions in CNS axonal density in vehicle-treated rats, compared to control rats at 3 weeks (Fig. 5A and C) and 8 weeks (Fig. 5B and D) after the injection. In particular, at 8 weeks after injection, a significant number of neurons exhibited complete axonal loss (Fig. 5D). In contrast, a higher number of axons with normal morphology were observed in EMSCs (Fig. 5E–H) and PMECs (Fig. 5I–L) -treated rats. Axonal loss scores (Fig. 5S and T) also confirmed the axonal-protective effects of EMSCs and PMECs treatments, with no difference between the axonal loss scores in EMSCs and PMECs-treated rats (Fig. 5S and T). Moreover, within the engrafted EMSCs (Fig. 5M and N) and PMECs (Fig. 5O and P), we also found a few axon-like fibers revealed by silver staining, although did not observe new neuronal growth (GFP+) in the cortex or spinal cord where neural injuries occurred.

### EMSCs and PMECs treatments reversed the decrease of BDNF and CNTF in CNS, increased the expression of growth-associated protein GAP-43, and reduced apoptosis and neuronal loss in EAE

One theory that may explain the therapeutic effects seen by MSC treatment in MS is their neuroprotective effects, which have been shown to increase the expression of neurotrophins and decrease the expression of pro-apoptotic factors. Therefore, we examined the expression of BDNF, CNTF, GAP-43, and caspase-3 to elucidate the possible neuroprotective effects of EMSCs and PMSCs following transplantation. Consistent with the results of immunofluorescence staining (Figure S5–S7), Western blots (Figure S8) showed that, compared with controls, EAE-induction slightly increased the expression of growth-associated protein GAP-43, whilst EMSCs and PMSCs treatment significantly increased expression (Figures S5 and S8A–C). Moreover, EAE induction remarkably decreased the expression of BDNF and CNTF in the CNS compared with controls, however, EMSCs and PMSCs treatment maintained expression at baseline levels compared to controls (Figure S8D–I). In vehicle-treated EAE rats, the expression of active caspase-3, an enzyme involved in apoptosis, was significantly increased (Figure S8J–L), while the expression of the neuronal marker NF-200 was evidently down regulated (Figures S6 and S8M–O). Down regulation of NF-200 was reversed by EMSCs and PMSCs transplantation.

Caspase-3 immunofluorescence staining showed that in vehicle-treated EAE rats, the expression of caspase-3 was up regulated in large, multipolar motor neurons in the spinal cord anterior horn and the pyramid-shaped motor neurons of the pre-central gyrus compared to those in the control group. Treatment with EMSCs and PMSCs at both 3 weeks and 8 weeks post-injection reversed this observation (Figure S6). NF-200 immunofluorescence staining (Figure S7) and Nissl staining (Figure S9) revealed visible neuronal loss was in the CNS of the vehicle-treated EAE rats, especially at 8 weeks after injection when compared to controls. Nevertheless, in EMSCs and PMSCs-treated EAE rats, a larger number of neurons were present in the anterior horn of the spinal cord and in the brain cortices.

### EMSCs and PMSCs Treatment Alleviated Reactive Astrocyte Proliferation and Reactive Gliosis in EAE Rats

**Figure 6:**
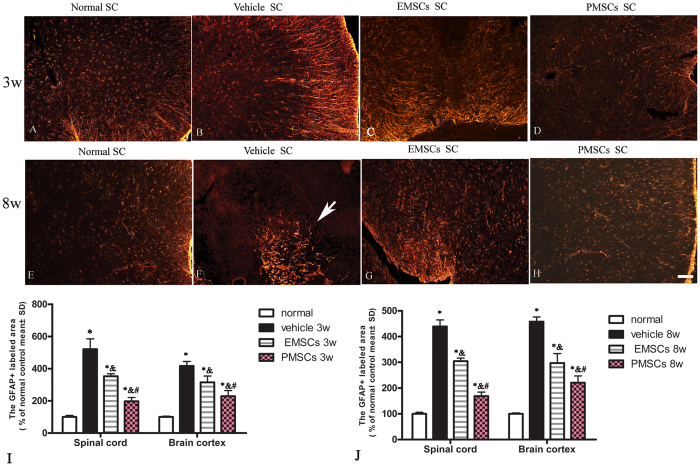
EMSCs and PMSCs treatments alleviated reactive astrocyte proliferation and reactive gliosis in EAE rats. EMSCs and PMSCs treatments inhibit reactive gliosis in EAE rats (**B,F**) at both 3 (**B–D**) and 8 weeks (**E–H**) post-injection. Arrow in F showed typical gliosis scar. TRIFC-conjugated red immunofluorescence indicate GFAP staining, (SC) Transverse sections through the anterior horn of the lumbar spinal. Scale bar = 100 μm (**I–J**): Quantification of GFAP^+^ cells, data are represented as mean ± SD. n = 5, degrees of freedom = 4. *P < 0.01 *Vs* Normal group (3w PI, in SP, Vehicle group: F = 0.02, P < 0.0001; EMSCs group: F = 0.24, P < 0.0001; PMECs group: F = 0.38, P < 0.0001. In BC, Vehicle group: F = 0.05, P < 0.0001; EMSCs group: F = 0.04, P = 0.0003; PMECs group: F = 0.02, P = 0.0001. 8w PI, in SP, Vehicle group: F = 0.06, P < 0.0001; EMSCs group: F = 0.16, P < 0.0001; PMECs group: F = 0.27, P < 0.0001. In BC, Vehicle group: F = 0.04, P < 0.0001; EMSCs group: F = 0.02, P = 0.0003; PMECs group: F = 0.009, P = 0.0001). ^&^P < 0.01 *Vs* Vehicle group (3w PI, In SP, EMSCs group: F = 10.65, P < 0.0001; PMECs group: F = 17.06, P = 0.002. In BC, EMSCs group: F = 0.69, P < 0.0001; PMECs group: F = 0.42, P < 0.0001. 8w PI, In SP, EMSCs group: F = 2.85, P < 0.0001; PMECs group: F = 4.81, P < 0.0001. In BC, EMSCs group: F = 0.48, P < 0.0001; PMECs group: F = 0.24, P < 0.0001). ^#^P < 0.05 *Vs* EMSCs group (3w PI, In SP, PMSCs group: F = 0.62, P < 0.0001; In BC, PMSCs group: F = 0.6, P = 0.005. 8w PI, In SP, PMSCs group: F = 1.69, P < 0.0001; In BC, PMSCs group: F = 1.97, P = 0.003).

Gliosis following inflammation is a hallmark of neural degeneration. To assess the effect of EMSCs and PMSCs treatment on EAE-induced reactive gliosis, we examined the expression of GFAP, a marker of astrocytes, by immunofluorescence labeling and Western blotting. At 3 weeks post-injection, immunofluorescent staining showed proliferation of astrocytes (Fig. 6B) and a visible glia scar at 8 weeks after injection (Fig. 6F) in vehicle-treated EAE rats compared to control rats (Fig. 6A). Conversely, GFAP expression and astrocyte proliferation were significantly reduced in EMSCs and PMSCs-treated groups at both 3 (Fig. 6C,D) and 8 weeks post-injection (Fig. 6G and H) in both the lumbar spinal cord and brain cortex. The results of Western blotting were consistent with those of our earlier morphological observations (Figure S7K–P).

### Transplanted EMSCs and PMSCs migrate and infiltrate into parenchymal of CNS and express neural–glial lineage markers

**Figure 7:**
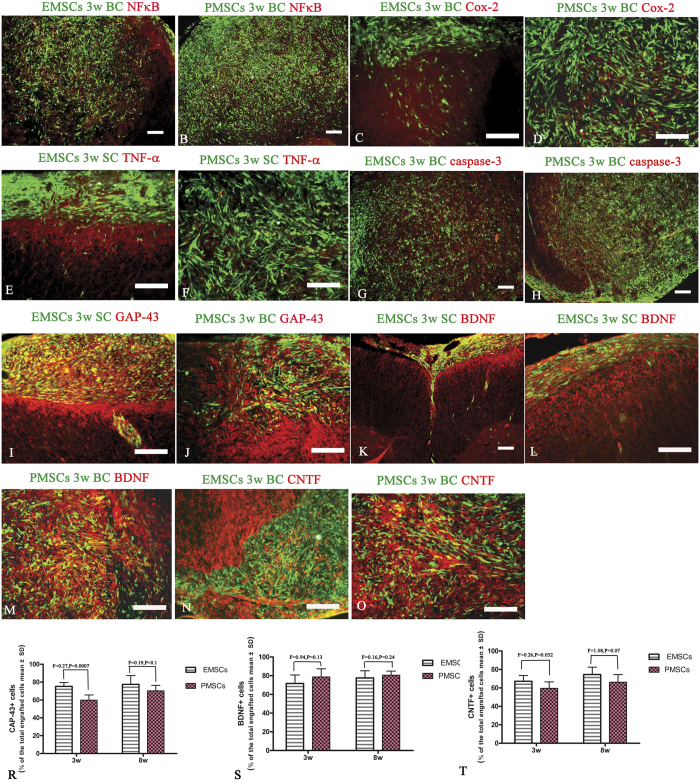
Engrafted EMSCs and PMECs fail to express pro-inflammatory factors and an apoptosis related enzyme, but express a growth-associated protein. The transplanted EMSCs and PMSCs (GFP-conjugated, green) do not express pro-inflammatory factors NF-κB (**A,B**), COX-2 (**C,D**), TNF-α (**E,F**), and active caspase-3 (**G**–**H**). However, the expressions of growth-associated protein GAP-43 (**I,J**) and neurotrophic factors BDNF (**K,L**) and CNTF (**M,N**) are found in both EMSCs and PMECs grafts. NF-kB, COX-2, TNF-α− GAP-43, BDNF and CNTF-TRIFC-conjugated immunofluorescence staining (red), the co-expression of GFP was showed as yellow. Scale bar = 100 μm. (SC) Transverse sections through the anterior horn of the lumbar spinal. (BC) Coronal sections of the brain cortex. (**R**–**T**) The quantification of GAP-43, BDNF and CNTF expression in engrafted EMSCs and PMSCs (Data are represented as mean ± SD. n = 5, degrees of freedom = 4).

**Figure 8:**
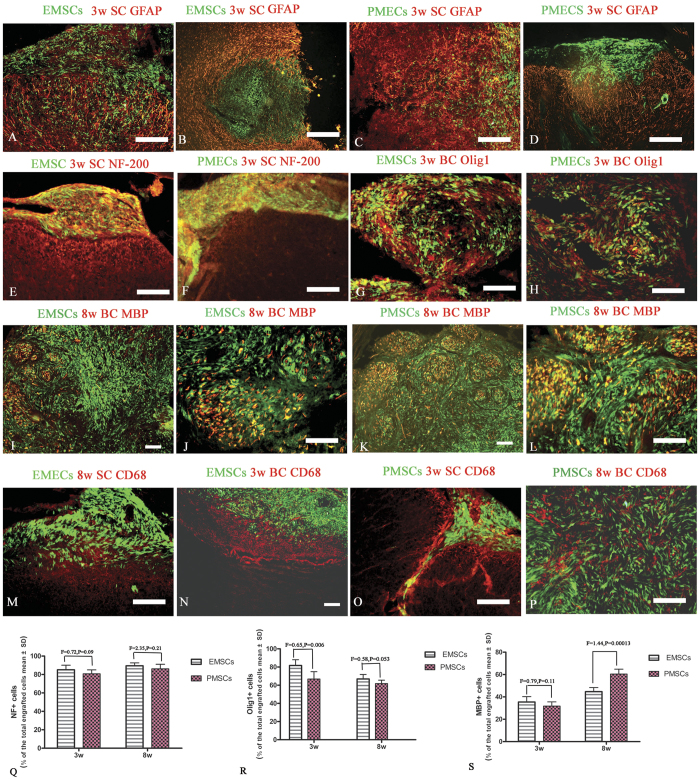
Transplanted EMSCs and PMSCs migrate and infiltrate into parenchymal of CNS and express neural–glial lineage markers. The immunofluorescence staining of the astrocyte specific marker GFAP (red, **A–D**), neuron specific marker NF-200 (red, **E,F**), oligodendrocyte precursor cells specific marker Olig1 (red, **G,H**), microglia/macrophage specific marker CD68 (green, **M–P**) and myelin marker protein, MBP on engrafted EMSCs and PMSCs (GFP-conjugated, green, **I–L**). Engrafted EMSCs and PMSCs rarely express GFAP, and GFAP positive astrocytes are mainly located in the circumjacent area of grafts (**A,B,D**). The glial fibers surround the margin of grafts, but engrafted MSCs could infiltrate and pass through these fibers to invade into the parenchyma of CNS (**C**). Both EMSCs and PMSCs express NF-200 (**E,F**), Olig1 (**G,H**) and MBP (**I–L**), however, Olig1 expression mainly appears at 3 weeks post-injection, and MBP mainly at 8 weeks post-injection. Also at 8 weeks post-injection, the transplanted EMSCs and PMECs form cellular mass in the parenchyma of CNS. Obviously MBP expression are revealed within these cellular mass expect in the central part (**I–L**). Moreover, engrafted EMSCs and PMSCs rarely express CD68, with CD68 positive microglia/macrophages mainly surrounding the graft (**M**–**O**) and localized in the central part of the cellular masses (P). Scale bar = 100 μm. (SC) Transverse sections through the anterior horn of the lumbar spinal. (BC) Coronal sections of the brain cortex. (**Q–S**) The quantification of NF-200, Olig1 and MBP expressions in engrafted EMSCs and PMSCs (Data are represented as mean ± SD. n = 5, degrees of freedom = 4).

To test whether transplanted EMSCs and PMSCs could migrate, infiltrate, and integrate into the cerebral cortex and spinal cord, we transplanted GFP-conjugated MSCs and examined the expression of multiple neural markers to assess their fate. Engrafted MSCs expressed GAP-43, BDNF, CNTF (Fig. 7I–N), as well as neural–glial lineage markers NF-200, and Olig1 (Fig. 8E–H). Expression of MBP was detectable at 8 weeks post-injection (Fig. 8I–L). However, the pro-inflammatory factors NF-kB, COX-2, TNF-α (Figs 5F–7A), and pro-apoptotic active caspase-3 (Fig. 7G,H) were lowly expressed in both EMSCs or PMECs. Moreover, engrafted EMSCs and PMSCs lowly expressed CFAP and CD68. GFAP positive astrocytes were mainly located in the circumjacent areas of grafts (Fig. 8A,B and D), and glial fibers surrounded the margin of grafts. However, engrafted MSCs passed through these fibers to infiltrate into the parenchyma of CNS (Fig. 8C) and formed cellular masses (Figure S8J and K, Figs 7 and 8). MBP expression was demonstrated within these cellular masses, except in the central areas (Fig. 8I–L) invaded by CD68 positive microglia/macrophages (Fig. 8P).

## Discussion

MSCs transplantation has emerged as an attractive therapy for MS due to ease of expansion and immunomodulatory and neuroprotective effects of MSCs, and demonstrated fewer side effects than other therapies^12^,^13^. Furthermore, MSCs are known to be associated with a level of immunoprivilege allowing allogeneic transplantation, and an ability to migrate from the blood to tissue allowing intravascular administration^22^. Compared with adult MSCs, EMSCs from embryonic stem cells have a higher propensity to expand, but their origin is limited by ethical concerns. However, PMSCs from placental cells may have a similar potential as EMSCs, and avoid ethical problems associated with ESC^23^. In the current study, we compared the efficacies of EMSCs and PMSCs in an EAE rat model. Our results suggest that the two types of MSCs have a similar capacity in ameliorating the MS-like phenotypes seen in EAE rats. In spinal cord, PMSCs even have a higher anti-inflammatory effect than EMSCs (Fig. 1). These characteristics of PMSCs may originate from endothelial stem cells or hematopoietic stem cells in the placenta during isolation^23^. So far, there is only one published report on the application of PMSCs in an EAE mouse model with few pathological studies published^15^. Therefore, we are the first to show that PMSCs function in a similar way to EMSCs in treating MS in the EAE rat model, specifically by up-regulating anti-inflammatory and neuroprotective factors and down-regulating pro-inflammatory and neurotoxic signals.

Another issue commonly addressed in MS studies is the cell fate of MSCs following transplantation. There is currently limited evidence to suggest whether transplanted cells migrate, infiltrate, integrate, and replace the degenerated neural cells. Variability in results from previous reports may arise from different experimental conditions, such as cell density and the severity of inflammation in the model used^24^. Compared with a similar approach used previously^24^, we administered a medium density of cells and a medial level of inflammation in our model. Our results demonstrated that although GFAP positive astrocytes extended into glial fibers surrounding the circumjacent area of the grafts, engrafted MSCs infiltrated and invaded through these fibers into the parenchyma of CNS and formed a cellular mass similar to other previously published observations^25^,^26^. In the center of cellular masses, local invasion of CD68 positive inflammatory cells was detected. However, the absence of co-localization of GFP-conjugated MSCs and CD68 suggests that transplanted MSCs do not differentiate into inflammatory cells. Previous studies have shown the ability of MSCs to differentiate into neural-like and glial-like cells *in vitro* and their potential for neuroregeneration in models of neuronal injures^27^,^28^. In our study, engrafted EMSCs and PMSCs did not express GFAP, but expressed the neuronal marker NF-200, oligodendroglia precursor cells marker Olig1 and oligodendrocyte marker MBP, which implies that MSCs have the potential of differientiating into oligodendrocytes and neurons. However, we failed to locate migrated EMSCs or PMSCs at the injured anterior horn of spinal cord or the motor cortex. We only detected several axon-like fibers without typical axons and myelin within the cellular mass of MSCs. All these observations suggest that engrafted MSCs may produce therapeutic benefits by transmigrating and differentiating into neural cells without engrafting into the injured tissues. The therapeutic effects of EMSCs or PMSCs are more likely to depend on autocrine and paracrine mechanisms to mediate the release of growth factors, anti-inflammatory cytokines, and anti-apoptotic signals, thus creating a favorable environment for the survival of neurons and maintenance of myelin and axons.

The administration methods of MSCs transplantation may affect observed results between studies. The comparison of intravenously (IV) and ICV in an MSC transplantation study suggests that ICV injection, which directly injects MSCs into cerebrospinal fluid (CSF), is superior to IV since it results in a more localized immunomodulatory outcome and a significant decrease in lymphocytic infiltrate^28^. Moreover, intrathecal (ITH) administration of BMSCs has also been considered an option performed in pilot clinical trials^29^. Therefore, we chose ICV and ITH injection as the route of transplantation in our study.

In the majority of studies utilizing MSCs transplantation in MS, therapeutic effects have been attributed to immunomodulation^30^,^31^, anti-inflammatory^32^, and neuroprotective^33^ properties. Our results confirmed these initial suggestions. We observed the down regulation of pro-inflammatory cytokines TNF-α, IFN-γ, IL-2, transcription factors COX-2 and NF-κB, and decreased pro-apoptotic enzyme active caspase-3, but increased the expression of the anti-inflammatory cytokines TGF-β and IL-4. An anti-inflammatory microenvironment is beneficial for preserving myelin integrity and reducing axonal loss and preventing apoptosis of neurons and oligodendrocytes. MSCs can also provide a source of stem cells with the potential to migrate into inflamed CNS tissue and differentiate into neurons and oligodendrocytes, as well as neurotrophic factors capable of stimulating endogenous repair pathways^34^. In our study, both Western blot and immunofluorescence staining demonstrated that the EMSCs and PMSCs transplanted groups exhibited up-regulated expression of BDNF and CNTF, two neurotrophic factors normally expressed in the cerebral cortex and spinal cord^35^,^36^. CNTF can promote neuronal survival and reduce TNF-α induced cell death in oligodendrocytes^37^. Furthermore, BDNF can maintain the survival of existing neurons, and promote the growth and differentiation of new neurons and synapses. High levels of BDNF have been reported to play a key role in neuroprotection^38^. Moreover, the increased expression of GAP-43, a protein considered crucial for axon regeneration, has been shown to prevent neural cell death and promote axonal regeneration, and was demonstrated to be expressed in our EMSCs and PMECs treated groups^39^. However, inflammatory related factors such as NFκB, TNF-α, and COX-2, which all act as tissue-damaging agents through regulating the expression of genes related to cytokines and consequently leading to oligodendrocyte and neuron apoptosis, were hardly detected in transplanted MSCs. All of these factors may affect neural cells within the CNS of transplant recipients as well as the transplanted MSCs themselves, reducing the expression of active caspase-3.

In conclusion, our results showed that PMSCs, similar to EMSCs, have the ability to migrate into inflamed CNS tissue, differentiate into cells expressing neural–glial lineage markers, and contribute to a variety of anti-inflammatory effects. Due to ease of access, lack of ethical conflicts, non-invasive acquisition, and abundance, PMSCs may be a useful source of transplantation for MS to replace EMSCs. However, the invasion of inflammatory cells into the graft may weaken the survival of transplanted cells, a combination of agents which can further inhibit inflammatory cell infiltration may further improve the effects of PMSCs transplantation. We are currently undertaking further research to establish such treatment regimes.

## Methods

### Isolation and Culture of Placental MSCs from GFP-rats

Placental MSCs were extracted from the placenta as previously described^15^. The placentas of healthy 18-day pregnant naïve/GFP-rats were collected after Caesarean section. The minced tissue was transferred to a 50 mL centrifuge tube containing DMEM (Invitrogen, Carlsbad, CA, USA) plus 0.1% bovine serum albumin (BSA) (Sigma-Aldrich, St. Louis, MO, USA) and digested for 45 min at 37 °C with a combination of 0.1% collagenase type IV (Sigma-Aldrich, St. Louis, MO, USA), 200 μg/mL DNAse I (Worthington, Lakewood, NJ, USA), and 0.1% dispase (Invitrogen Carlsbad, CA, USA). The tissue was triturated every 10 min using 10 mL plastic serological pipettes. The resulting homogenate was successively filtered through a 100 μm Nytex mesh (BD Falcon, MD, USA), placed in a standard laboratory funnel, and then centrifuged at 800 g for 20 min. The cell pellet was resuspended twice and plated on dishes coated with human placental fibronectin (100 ng/mL; Sigma-Aldrich, St. Louis, MO, USA) at a density of 1 × 10^6^ cells/80 cm^2^ (i.e., 1 × 10 cm dish) in DMEM (Invitrogen, Carlsbad, CA, USA) containing 10% fetal bovine serum (FBS) (Biological Industries, Beit Haemek, Israel), 1:100 non-essential amino acids (Biological Industries, Beit Haemek, Israel), 55 μM beta-mercaptoethanol (Sigma-Aldrich, St. Louis, MO, USA), 10 μg/mL ciprofloxacin (Bayer, Leverkusen, Germany), and 10 μg/mL amphotericin B (Invitrogen, Carlsbad, CA, USA).

GFP-conjugated Embryonic Stem Cell-Derived Mesenchymal Stem Cells (EMSCs) was purchased from Cyagen Biosciences and cultured in Murine Mesencult medium (Stem Cell Technologies, Vancouver, BC). Cells were kept in a humidified 5% CO_2_ incubator at 37 °C, and the medium were refreshed every 3 to 4 days, for 4 to 5 weeks.

### Characterization of MSCs

To test the differentiation potential of PMECs, a cocktail of 10 ng/mL EGF and 10 ng/mL bFGF was added into medium of cultured naïve PMECs passage 3. After 24 hours, the cultures were washed with PBS and fixed with 4% paraformaldehyde for analysis of cell determinant markers. The expressions of surface antigens CD44 were used as a marker for MSCs. Multiple specific markers (GFAP for astrocytes, CD68 for microcytes/macrophages, Olig1 for oligodendrocytes and NF-200 for neurons) were double stained with CD44 to evaluate the differentiation potentials of MECs.

### Animals and EAE Induction

A total of 56 adult male Lewis rats (9–10 weeks, 200–250 g) were obtained from Zhejiang University Laboratory Animal Services Center. Of these, 8 were used as healthy controls and the remaining 48 were randomly assigned into 3 groups: one vehicle-treated group, one EMSCs transplanted group, and one PMSCs transplanted group, n = 16/group (n = 8/time point, with 5 used for histological assessments and the other 3 used for ELISA/Western blotting assays). Experiments were performed in accordance with National Institutes of Health (NIH) Guidelines for the Care and Use of Laboratory Animals, with approval from the animal ethics committee of Zhejiang University.

EAE was induced in EMSCs, PMSCs transplanted and vehicle-treated rats by subcutaneously injecting 0.2 mL of a 1:1 mixture of guinea pig spinal cord homogenate (GPSCH) and complete Freund’s adjuvant (CFA), containing 0.5 mg of heat-inactivated *Mycobacterium tuberculosis* (Difco Laboratories, Detroit, MI). Rats in the normal control group were injected with CFA emulsified 1:1 with 0.9% saline. Immediately after CFA injection, and again 24 h later, rats received pertussis toxin (300 ng, intraperitoneally, i.p. Sigma, St. Louis, MO) in 0.1 mL phosphate-buffered saline (PBS), and this process was repeated after 48 h^40^,^41^.

Animals were assessed for clinical signs of disease each week post-immunization (pi) until sacrifice. Severity of the signs was assessed using a scale ranging from 0 to 5: grade 0 = no signs, grade 1 = partial loss of tail tonicity, grade 2 = total loss of tail tonicity, grade 3 = unsteady gait and mild paralysis, grade 4 = hind limb paralysis and incontinence, and grade 5 = moribund or death^42^. Induction of the EAE model was considered successful if rats were assigned a score that exceeded 2, and animals that did not obtain a score of 2 were considered unsuccessful and excluded. Disease severity was assessed until sacrifice and no animals were scored above grade 4. In the normal control group, scores remained as grade 0.

### Stem cell transplantation

Ten days after EAE induction, rats received bilateral ICV injections of 1 × 10^6^ PMSCs, EMSC or phosphate-buffered saline (PBS) in a volume of 2 μL using a Hamilton 10 μL syringe with a 26-gauge needle. The coordinates of the injections were as follows: AP + 0.6 mm, ML ± 0.7 mm, and V −3 mm, from Bregma based on the mouse stereotaxic atlas (Paxinos and Watson). Two hours before transplantation, the GFP+ labeled cells were detached from the dishes by using a cell lifter, collected by centrifugation at 1000 × g for 4 min, and resuspended in 1 mL culture medium. After cell counting and viability assessment with Trypan blue in a hemacytometer, the cell suspension was centrifuged a second time and resuspended in a smaller volume to give a density of 1 × 10^6^ viable cells/μL.

For ITH transplantation, the vertebrae were carefully separated using two fine tweezers in order to reveal the lumbar spinal cord (L4–L5). EMSCs, PMECs (1 × 10^6^ cells) or saline were injected intrathecally with a glass micropipette connected to the nanoinjector (Nanoinject II; Drummond Scientific Company, Broomall, PA, USA) at the rate of 1 μL/minute for a total volume of 4 μL.

### Neurophysiological testing

Cortical somatosensory evoked potentials (c-SEPs) were recorded at 3 weeks, the peak stage of MSCs transplanted groups, and 8 weeks after immunization, the recovery stage in vehicle-treated rats, for five rats in each group immediately prior to sacrifice. Rats were fixed in a stereotaxic frame and surgical procedures were performed as previously described^19^. An isolated constant current stimulator (Digitimer, Welwyn Garden City, UK) was used to deliver positive current pulses with a sufficiently large amplitude (15 V) and duration (40 ms) for producing a maximum SEP (averaged over 30 stimuli)^19^,^20^,^21^. SEPs were amplified, filtered, digitally converted, and stored for post-hoc analysis. Statistical analyses were performed on values obtained from three series of stimulations. Peak positive and negative values were measured, and results were expressed as the mean ± standard deviation (SD) of voltage amplitude (μV) and latency (ms).

Cortical motor evoked potentials (c-MEP) were recorded at the same time points as the c-SEPs. Following anesthesia, a midline incision was made on the scalp, and the tissues underneath were cleaned and the cranium exposed. Screw electrodes were implanted to a depth of 0.75 mm over the primary somatomotor cortical areas, lightly contacting but not pressuring or puncturing the dura mater. An active needle electrode was inserted into the muscle of the hindlimb, and a reference electrode was inserted under the skin, 2 mm from the screw electrode. The somatobrain cortex was stimulated at 10 Hz with trains of 10–25 pulses, which evoked visible contralateral hindlimb movement, and signals were averaged for obtaining the c-MEP^43^,^44^.

### Perfusion and tissue processing

Animals of all treatment groups were sacrificed at 3 weeks and 8 weeks post-injection (five animals per time point in each group). Half of the brain and spinal cord tissues from each animal were prepared for histological assessment and immunohistological and immunofluorescent staining, and the remainder of the central nervous tissue were examined by transmission electron microscope analysis. Perfusion and tissue processing were performed as previously described^40^,^41^,^42^.

### Histological assessment

Digital photomicrographs were obtained at 200x magnification in three visual fields/per section. Nissl staining with Cresyl violet was employed to assess inflammation and neuronal survival. The severity of inflammatory cell infiltration was scaled as follows^21^: 0, no inflammation; 1, cellular infiltration only around blood vessels and meninges; 2, mild cellular infiltration in the parenchyma (1–10 cells/section); 3, moderate cellular infiltration in the parenchyma (11–100 cells/section); and 4, severe cellular infiltration in the parenchyma (100+ cells/section). Neuronal counts were restricted to cells with a well-defined nucleolus and a cell body that displayed adequate amounts of endoplasmic reticulum.

Myelin basic protein (MBP) immunofluorescence staining was used to evaluate the degree of demyelination. Demyelination was scored as follows^45^: 0, normal white matter; 1, rare foci of demyelination; 2, a few areas of demyelination; 3, confluent perivascular or subpial demyelination; 4, massive perivascular and subpial demyelination involving one half of the spinal cord, with the presence of cellular infiltrates in the CNS parenchyma; and 5, extensive perivascular and subpial demyelination involving the whole cord section, with the presence of cellular infiltrates in the CNS parenchyma.

Bielschowsky silver staining was performed to estimate axonal loss^40^,^41^,^42^, which was assessed using the following scale^46^: 0, no loss; 1, a few foci of superficial loss involving less than 25% of tissue; 2, foci of deep axonal loss, encompassing over 25% of tissue; and 3, diffuse and widespread axonal loss.

### Immunohistochemical and immunofluorescence staining

The sections used for immunofluorescence staining were incubated with primary monoclonal mouse anti-MBP antibody (1:500, Abcam, Cambridge, MA), anti-GAP43 and anti-CD68/ED1 (1:100; Santa Cruz, Dallas, TX) antibodies and polyclonal rabbit anti-activated caspase-3 (1:500; Cayman Chemical, Ann Arbor, MI), anti-NF200 (1:500, Abcam, Cambridge, MA), anti-GFAP (1:200, Thermo Fisher Scientific Waltham, MA), anti-Olig1 (1:500; Abcam, Cambridge, MA), anti-CNTF (1:500, Abcam, Cambridge, MA), anti-NF-kB p65 (1:500, Abcam, Cambridge, MA) antibodies and anti-BDNF(1:500, Abcam, Cambridge, MA) antibodies overnight at 4 °C. The sections were then washed with phosphate-buffered saline (PBS) and incubated with 1:200 TRITC (rhodamine)-conjugated secondary antibodies for 1 h at 37 °C (Invitrogen, Carlsbad, CA). The sections were finally coverslipped with Antifade Gel/Mount Aqueous Mounting Media (Southern Biotech, Birmingham, AL).

Five sections from the brain cortex and anterior horns of the spinal cord for each animal were randomly selected and images were photographed under 200x magnification in three vision fields per section. GFAP, MBP, CD68-immunoreactive areas were analyzed with NIH image software, and the numbers of GAP43, caspase-3, NF-200, Olig1, CNTF, NF-kB p65, BDNF-labeled cells were counted.

### Electron microscopy

Processing for electron microscopy was performed as described previously^40^,^41^,^42^. Images were captured first at low resolution and then at higher magnification in a variety of regions of the brain cortex and lumbar spinal cord.

### Quantification of cytokine levels by ELISA

Peripheral blood samples were collected from rats killed by decapitation at 3 and 8 weeks after immunization (n = 3 per time point/group). ELISAs for IL-2, IL-4, TNF-α (all from Abcam, Cambridge, MA), and IFN-γ (BioLegend Inc., San Diego, CA) were performed as previously described^40^,^41^,^42^. Optical density was measured at 450 nm and analyzed using GraphPad Prism 4 (GraphPad Software, Inc., San Diego, CA).

### Western blotting

Rats were sacrificed by decapitation at 3 weeks and 8 weeks post-immunization (n = 3 per time point per group), and whole brain cortex tissue and 10-mm lumbar spinal cord segments were prepared for Western blotting. Processing for Western blotting was performed as described previously^40^,^41^,^42^. Briefly, equal amounts (40 μg) of total protein extracts were prepared, and each sample was separated by Tris–glycine SDS-PAGE on 12% acrylamide gels at 4 °C and then transferred to a polyvinylidene fluoride membrane (Millipore, Billerica, MA). Each membrane was incubated for 12 h at room temperature with primary rabbit polyclonal anti-NF-kB p65 (1:1000, Abcam, Cambridge, MA), anti-activated caspase-3 (1:1000; Cayman Chemical, Ann Arbor, MI), anti-NF200 (1:1000, Abcam, Cambridge, MA), anti-GFAP (1:800, Thermo Fisher Scientific Waltham, MA), anti-CNTF (1:1000, Abcam, Cambridge, MA), anti-BDNF(1:1000; Abcam, Cambridge, MA) and anti-Olig2 (all 1:500; Abcam, Cambridge, MA) antibodies, mouse anti-MBP antibody (1:1000, Abcam, Cambridge, MA), anti-GAP43 and anti-CD68/ED1 (1:1000; Santa Cruz, Dallas, TX) antibodies. After incubation with the corresponding secondary horseradish peroxidase (HRP)-conjugated antibody (1:1000, Santa Cruz), Western blots were visualized using an ECL Plus Detection System, followed by imaging and quantification of protein bands using Bio-Rad Quantity One 1-D software (Bio-Rad, Hercules, CA). To normalize protein bands to a gel loading control, membranes were washed in TBST and re-probed with rabbit anti-β-actin (1:5000, Abcam, Cambridge, MA), followed by incubation with HRP-conjugated goat anti-rabbit antibody (1:5000, Santa Cruz, Dallas, TX) and ECL detection. For the negative control, the primary antibody was omitted.

### Statistical analysis

Differences between protein levels were analyzed with two-way ANOVA, followed by post-hoc Tukey *t*-tests. Data were analyzed using SPSS 13.0 software and P-values of less than 0.05 were considered statistically significant. All statistical graphs were created with GraphPad Prism Version 4.0 (GraphPad Prism Software, Inc., San Diego, CA).

## Additional Information

**How to cite this article**: Jiang, H. *et al*. Amelioration of experimental autoimmune encephalomyelitis through transplantation of placental derived mesenchymal stem cells. *Sci. Rep.* **7**, 41837; doi: 10.1038/srep41837 (2017).

**Publisher's note:** Springer Nature remains neutral with regard to jurisdictional claims in published maps and institutional affiliations.

## Supplementary Material

Supplemental Information

## Figures and Tables

**Table 1:** EMSCs and PMSCs transplant reduce c-SEP and MEP latencies, increase c-SEP and MEP amplitudes at both 3 and 8 weeks post-injection.

3 weeks	C-SEP Latency (ms)		
Group	N	P	Wave amplitude (μV mean ± SD)
Normal	13.95 ± 0.66** (F = 0.32, P = 0.0004)	15.65 ± 0.89** (F = 0.33, P < 0.0001)	2.6 ± 0.5** (F = 183.63, P = 0.0003)
Vehicle	17.1 ± 1.17	24.57 ± 1.54	0.44 ± 0.04
EMSCs	14.47 ± 0.76** (F = 2.36, P = 0.0014)	18.18 ± 0.61** (F = 0.16, P = 0.0002)	1.99 ± 0.97** (F = 6.82, P < 0.0001)
PMSCs	15.27 ± 0.58* (F = 4.08, P = 0.045)	18.48 ± 1.13** (F = 1.85, P < 0.0001)	1.59 ± 0.55** (F = 222.53, P = 0.0048)
**3 weeks**	**MEP Latency (ms)**		
**Group**		**Wave amplitude (μV mean ± SD)**	
Normal	5.54 ± 0.20** (F = 0.03, P < 0.0001)	3.38 ± 0.54** (F = 1165.8, P < 0.0001)	
Vehicle	12.17 ± 1.1	0.11 ± 0.02	
EMSCs	5.63 ± 0.34** (F = 0.11, P < 0.0001)	3.15 ± 0.89** (F = 3155.48, P = 0.0008)	
PMSCs	5.20 ± 0.21** (F = 0.04, P < 0.0001)	2.97 ± 0.33** (F = 436.28, P < 0.0001)	
**8 weeks**	**C-SEP Latency (ms)**		
**Groups**	**N**	**P**	**Wave amplitude (μV mean ± SD)**
Normal	14.36 ± 0.53** (F = 0.24, P = 0.0003)	18.04 ± 0.94** (F = 0.63, P = 0.0002)	2.14 ± 0.25** (F = 2.02, P < 0.0001)
Vehicle	17.29 ± 1.09	21.83 ± 1.18	0.65 ± 0.18
EMSCs	14.37 ± 1.02** (F = 0.87, P = 0.0012)	17.20 ± 1.19** (F = 1.014, P = 0.00013)	1.99 ± 0.8* (F = 20.51, P = 0.012)
PMECs	16.13 ± 0.63* (F = 0.33, P = 0.037)	19.22 ± 1.74* (F = 2.19, P = 0.012)	2.10 ± 0.65** (F = 13.73, P = 0.0026)
**8 weeks**	**MEP Latency (ms)**		
Groups		**Wave amplitude (μV mean ± SD)**	
Normal	5.52 ± 0.22** (F = 1.61, P < 0.0001)	4.28 ± 1.15** (F = 1433.37, P < 0.0001)	
Vehicle	12.26 ± 0.17	0.14 ± 0.03	
EMSCs	5.44 ± 0.64** (F = 13.6, P < 0.0001)	6.38 ± 1.76** (F = 3335.48, P < 0.0001)	
PMSCs	5.19 ± 1.31** (F = 56.98, P = 0.000014)	4.0 ± 1.38** (F = 2059.441, P = 0.00012)	

^*^P < 0.05 versus vehicle-treated EAE rats. ^**^P < 0.01 versus vehicle-treated EAE rats. N, negative deflection; P, positive deflection.
