# Supplementary material for: Amelioration of experimental autoimmune encephalomyelitis through transplantation of placental derived mesenchymal stem cells
Source: Sci Rep. 2017 Feb 10;7:41837. doi: 10.1038/srep41837 (PMC5301256; doi:10.1038/srep41837)
Supplement: Supplemental Information [file srep41837-s1.pdf]

Amelioration of experimental autoimmune encephalomyelitis through transplantation of placental derived mesenchymal stem cells

Hong Jiang[1], Yuanyuan Zhang[2#], Kewei Tian[2#], Beibei Wang[3], Shu Han[2*]

[1] Department of Electrophysiology, Sir Run Run Shaw Hospital, Medical College, Zhejiang University, Hangzhou, China

[2] Institute of Anatomy and Cell Biology, Medical College, Zhejiang University, Hangzhou, China

[3] Core Facilities, Zhejiang University School of Medicine, Hangzhou, China

[#]These authors contributed equally to this work.

***Correspondence to**: Dr. Shu Han

Institute of Anatomy and Cell Biology, Medical College, Zhejiang University, 866 Yuhangtang Road, Hangzhou 310058, China. **Tel.:** +86-571-88208160; **Fax:** +86-571-88208094; **Email:** han00shu@zju.edu.cn.

Supplemental figure legends

Figure S1. Differentiation of PMSCs cultured in vitro. PMSCs from passage 3 exhibits morphological characterization of different neural cell lines. (A-D) Astrocytes-like cells differentiate from PMECs. (E-H) Oligodendrocytes-like cells differentiate from PMECs. (I-L) Microcyte-like cells differentiate from PMECs. (M-P) Neuron-like cells differentiate from PMECs.

Figure S2. Both EMSCs and PMECs treatment reverse electrophysiological dysfunction (A-B): EMSCs and PMSCs treatments reduce the clinical severity of EAE in rats at 3 (A) and 8 (B) weeks post-injection, as measured by determining somatosensory-evoked potential (c-SEP) latencies and amplitudes (measured from peak to peak between negative deflection (N) and positive deflection (P). The amplitude of c-SEP is notably lower in vehicle-treated EAE rats and the latency is also significantly prolonged, while treatments with EMSCs and PMSCs effectively reversed these phenomena. (C-D): Motor-evoked potential (MEP) amplitude was also significantly lower in vehicle-treated EAE rats and latency is significantly prolonged at 3 (C) and 8 (D) weeks post-injection. Similarly, these phenomena are reversed following EMSCs and PMSCs treatments.

Figure S3. Compared with the vehicle-treated group, EMSCs and PMSCs treatments effectively reduce the expression of pro-inflammatory factors NF-kB (A-C), TNF-α(**D-F**), COX-2(G-I), but maintained the expression of oligodendrocyte marker MBP (**J-L**) as detected by Western blotting. Data are represented as mean ± SD. n=5, degrees of

freedom=n-1. **NFkB:** ***P<0.05*Vs* Normal group (3w Vehicle group: F=0.07, P<0.0001; EMSCs group: F=0.04, P=0.009; 8w PI, Vehicle group: F=0.33,P<0.0001; EMSCs group: F=0.26, P=0.0002) **&** P<0.01 *Vs* Vehicle group (3w PI, EMSCs group: F=0.57,P=0.007; PMECs group: F=4.19,P<0.0001. 8w PI, EMSCs group: F=0.78,P<0.0001; PMECs group: F=0.35, P<0.0001). # P<0.05 *Vs* EMSCs group (3w PI, PMSCs group: F=0.13, P=0.01; 8w PI, PMSCs group: F=0.44, P=0.048). **TNF-α:** ***P<0.05 *Vs* Normal group (3w Vehicle group: F=1.76, P<0.0001; EMSCs group: F=0.69, P<0.0001. PMECs group: F=0.65, P=0.0002; 8w PI, Vehicle group: F=0.03, P<0.0001; EMSCs group: F=0.1, P=0.0007. PMECs group: F=0.82, P=0.004) **&** P<0.01 *Vs* Vehicle group (3w PI, EMSCs group: F=0.39, P<0.0001; PMECs group: F=0.37, P<0.0001. 8w PI, EMSCs group: F=3.05, P<0.0001; PMECs group: F=26.23, P<0.0001). # P<0.01 *Vs* EMSCs group (8w PI, PMSCs group: F=0.11, P=0.0003). **COX-2**: ***P<0.05 *Vs* Normal group (3w Vehicle group: F=0.3, P<0.0001; EMSCs group: F=0.15, P=0.0001. PMECs group: F=0.27, P=0.0007; 8w PI, Vehicle group: F=0.05, P<0.0001; PMECs group: F=0.54, P<0.0001) **&** P<0.01 *Vs* Vehicle group (3w PI, EMSCs group: F=0.51, P<0.0001; PMECs group: F=0.89, P<0.0001. 8w PI, EMSCs group: F=1.56, P<0.0001; PMECs group: F=10.62, P<0.0001). # P<0.01 *Vs* EMSCs group (3w PI, PMSCs group: F=0.58, P<0.0001; 8w PI, PMSCs group: F=6.8, P=0.0009), **MBP:*** P<0.05 *Vs* Normal group (3w Vehicle group: F=0.4, P<0.0001; 8w PI, Vehicle group: F=1.25, P<0.0001; PMECs group: F=0.54, P<0.0001) **&** P<0.01 *Vs* Vehicle group (3w PI, EMSCs group: F=0.91, P<0.0001; PMECs group: F=0.53, P<0.0001. 8w PI, EMSCs group: F=0.07, P=0.0002; PMECs group: F=0.16, P<0.0001).

Figure S4. The expression of pro-inflammatory factors in response to EMSCs and PMSCs treatments. At 3 weeks post-injection, the expression of pro-inflammatory factors NF-kB (A–F) ***P<0.01 *Vs* Normal group (3w PI, in SP, Vehicle group: F=1.07, P<0.0001; EMSCs group: F=0.14, P<0.0001; PMECs group: F=0.23, P<0.0001. In BC, Vehicle group: F=157.26, P<0.0001; EMSCs group: F=286.87, P<0.0001; PMECs group: F=136.57, P<0.0001. 8w PI, in SP, Vehicle group: F=0.92, P<0.0001; EMSCs group: F=0.69, P<0.0001; PMECs group: F=0.34, P<0.0001. In BC, Vehicle group: F=0.08, P<0.0001; EMSCs group: F=0.12, P<0.0001; PMECs group: F=0.49, P<0.0001) **& P<0.01 *Vs* Vehicle group (3w PI, In SP, EMSCs group: F=0.13, P<0.0001; PMECs group: F=0.21, P<0.0001. In BC, EMSCs group: F=0.55, P<0.0001; PMECs group: F=1.15, P<0.0001. 8w PI, In SP, EMSCs group: F=0.37, P<0.0001; PMECs group: F=0.75, P<0.0001. In BC, EMSCs group: F=1.48, P<0.0001; PMECs group: F=6.16, P<0.0001), **COX-2 (G-L) ***P<0.01 *Vs* Normal group (3w PI, in SP, Vehicle group: F=0.05, P<0.0001; EMSCs group: F=0.28, P<0.0001; PMECs group: F=0.24, P<0.0001. In BC, Vehicle group: F=4.36, P<0.0001; EMSCs group: F=0.54, P<0.0001; PMECs group: F=0.7, P<0.0001. 8w PI, in SP, Vehicle group: F=0.22, P<0.0001; EMSCs group: F=0.15, P<0.0001; PMECs group: F=0.54, P<0.0001. In BC, Vehicle group: F=0.03, P<0.0001; EMSCs group: F=0.1, P<0.0001; PMECs group: F=0.06, P<0.0001) **&** P<0.01 *Vs* Vehicle group (3w PI, In SP, EMSCs group: F=0.17, P<0.0001; PMECs group: F=0.2, P<0.0001. In BC, EMSCs group: F=2.36, P<0.0001; PMECs group: F=3.07, P<0.0001. 8w PI, In SP, EMSCs group: F=1.45, P<0.0001; PMECs group: F=2.43, P<0.0001. In BC, EMSCs group: F=0.34, P<0.0001; PMECs group: F=0.55, P<0.0001) **and TNF-α(M–R) ***P<0.01 *Vs* Normal group (3w PI, in SP, Vehicle group: F=7.41, P<0.0001;

EMSCs group: F=7.89, P<0.0001; PMECs group: F=13.42, P=0.0007. In BC, Vehicle group: F=0.001, P<0.0001; EMSCs group: F=0.001, P<0.0001; PMECs group: F=0.0009, P<0.0001. 8w PI, in SP, Vehicle group: F=0.33, P<0.0001; EMSCs group: F=0.13, P<0.0001; PMECs group: F=0.48, P<0.0001. In BC, Vehicle group: F=7.08, P<0.0001; EMSCs group: F=10.51, P=0.0002; PMECs group: F=9.86, P=0.0002) **&** P<0.01 *Vs* Vehicle group (3w PI, In SP, EMSCs group: F=0.94, P<0.0001; PMECs group: F=1.81, P<0.0001. In BC, EMSCs group: F=1.02, P<0.0001; PMECs group: F=1.23, P<0.0001. 8w PI, In SP, EMSCs group: F=2.53, P<0.0001; PMECs group: F=0.68, P<0.0001. In BC, EMSCs group: F=1.49, P<0.0001; PMECs group: F=1.39, P<0.0001) **are increased in vehicle-treated rats relative to normal control rats, while expressions is alleviated in both EMSCs and PMSCs treated rats. TRIFC-conjugated immunofluorescent staining. Bar = 100μm. Data are represented as mean ± SD. n=5, degrees of freedom=4.**

Figure S5. EAE-induction slightly increases the expression of growth-associated protein GAP-43 in the early stages of disease, but expression decreased as EAE progressed. However, EMSCs and PMSCs treatments markedly up-regulated the expression of GAP-43. (A-N): TRIFC-conjugated red immunofluorescence indicate GAP-43 staining, Scale bar = 100 μm. (BC) Coronal sections of the brain cortex. Data are represented as mean ± SD. n=5, degrees of freedom=4. *****P<0.01 *Vs* Normal group (3w PI, in SP, Vehicle group: F=0.02, P<0.0001; EMSCs group: F=0.003, P<0.0001; PMECs group: F=0.004, P<0.0001. In BC, Vehicle group: F=0.15, P=0.0001; EMSCs group: F=0.03, P<0.0001; PMECs group: F=0.02, P<0.0001. 8w PI, in SP, Vehicle group: F=0.09, P<0.0001; EMSCs group: F=0.03,

P<0.0001; PMECs group: F=0.06, P<0.0001. In BC, Vehicle group: F=0.07, P<0.0001; EMSCs group: F=0.05, P<0.0001; PMECs group: F=0.03, P=0.0006.) **&** P<0.01 *Vs* Vehicle group (3w PI, In SP, EMSCs group: F=0.16, P<0.0001; PMECs group: F=0.19, P<0.0001. In BC, EMSCs group: F=0.11, P<0.0001; PMECs group: F=0.2, P<0.0001. 8w PI, In SP, EMSCs group: F=2.96, P<0.0001; PMECs group: F=0.63, P<0.0001. In BC, EMSCs group: F=0.8, P=0.0003; PMECs group: F=0.39, P=0.008).

Figure S6. EMSCs and PMECs treatments reduce neuronal apoptosis. In vehicle-treated EAE rats, the expression of active caspase-3 is markedly increased. This phenomenon could be reversed by EMSCs and PMSCs treatments, as shown by calculated TRIFC-conjugated (red color) immunofluorescence stained caspase-3 positive cells (scale bar = 100μm). (SC) Transverse sections through the anterior horn of the lumbar spinal. (BC) Coronal sections of the brain cortex. Data are represented as mean ± SD. n=5, degrees of freedom=4. *****P<0.01 *Vs* Normal group (3w PI, in SP, Vehicle group: F=0.03, P<0.0001; EMSCs group: F=0.04, P<0.0001; PMECs group: F=0.17, P<0.0001. In BC, Vehicle group: F=0.04, P<0.0001; EMSCs group: F=0.06, P=0.0002; PMECs group: F=0.2, P<0.0001. 8w PI, in SP, Vehicle group: F=0.2, P<0.0001; EMSCs group: F=0.43, P<0.0001; PMECs group: F=0.39, P<0.0001. In BC, Vehicle group: F=0.08, P<0.0001; EMSCs group: F=0.1, P<0.0001; PMECs group: F=0.3, P<0.0001) **&** P<0.01 *Vs* Vehicle group (3w PI, In SP, EMSCs group: F=1.4, P<0.0001; PMECs group: F=6.03, P<0.0001. In BC, EMSCs group: F=1.39, P<0.0001; PMECs group: F=4.8, P<0.0001. 8w PI, In SP, EMSCs group: F=2.15, P<0.0001; PMECs group: F=1.96, P<0.0001. In BC, EMSCs group: F=1.16, P<0.0001; PMECs group: F=3.49, P<0.0001.) #

P<0.01 *Vs* EMSCs group (8w PI, In SP, PMSCs group: F=1.1, P<0.0001; In BC, PMSCs group: F=3, P=0.0005).

Figure S7. EMSCs and PMSCs treatments alleviate neuronal loss in the spinal cord and cerebral cortex when compared with the vehicle-treated group. NF-200 immunofluorescence staining (red), scale bar = 100 μm. (SC) Transverse sections through the anterior horn of the lumbar spinal. (BC) Coronal sections of the brain cortex. Data are represented as mean ± SD. n=5, degrees of freedom=4. *****P<0.01 *Vs* Normal group (3w PI, in SP, Vehicle group: F=0.23, P<0.0001; EMSCs group: F=0.12, P<0.0001; PMECs group: F=0.12, P<0.0001. In BC, Vehicle group: F=0.04, P<0.0001; EMSCs group: F=0.02, P=0.00064; PMECs group: F=0.02, P=0.0001. 8w PI, in SP, Vehicle group: F=0.07, P<0.0001; EMSCs group: F=0.05, P<0.0001; PMECs group: F=0.48, P<0.0001. In BC, Vehicle group: F=0.04, P<0.0001; EMSCs group: F=0.02, P=0.0088; PMECs group: F=0.02, P=0.002) **&** P<0.01 *Vs* Vehicle group (3w PI, In SP, EMSCs group: F=0.51, P<0.0001; PMECs group: F=0.52, P<0.0001. In BC, EMSCs group: F=0.57, P<0.0001; PMECs group: F=0.56, P=0.0004. 8w PI, In SP, EMSCs group: F=0.75, P=0.00011; PMECs group: F=6.57, P<0.0001. In BC, EMSCs group: F=0.41, P<0.0001; PMECs group: F=0.48, P<0.0001.) # P<0.05 *Vs* EMSCs group (3w PI, In SP, PMSCs group: F=0.98, P=0.03; In BC, PMSCs group: F=1, P=0.004. 8w PI, In SP, PMSCs group: F=0.11, P=0.02).

Figure S8. EMSCs and PMECs treatments reverse the decrease of BDNF and CNTF in CNS, increase the expression of growth-associated protein GAP-43, and reduce

apoptosis related enzyme caspase-3 in EAE. (A-C): EAE-induction slightly increases the expression of growth-associated protein GAP-43 compared with healthy controls, but EMSCs and PMSCs treatments markedly increased expression. *P<0.01 *Vs* Normal group (3w Vehicle group: F=1.85, P=0.0009; EMSCs group: F=0.33, P<0.0001; PMECs group: F=0.65, P<0.0001. 8w PI, Vehicle group: F=0.15, P=0.0002; EMSCs group: F=0.16, P<0.0001; PMECs group: F=0.09, P<0.0001.) **&** P<0.01 *Vs* Vehicle group (3w PI, EMSCs group: F=0.18, P<0.0001; PMECs group: F=0.35, P<0.0001. 8w PI, EMSCs group: F=1.06, P=0.0005; PMECs group: F=0.62, P=0.0002) (D-I): EAE induction remarkably decreased the expression of BDNF and CNTF in CNS as compared with healthy controls, and EMSCs and PMSCs treatments maintained expression of these markers. BDNF: *P<0.05*Vs* Normal group (3w Vehicle group: F=4.62, P<0.0001;EMSCs group: F=0.56, P=0.003; PMECs group: F=0.13, P=0.02. 8w PI, Vehicle group: F=2.97, P=0.004; EMSCs group: F=8.87, P<0.0001; PMECs group: F=4.12, P<0.0001) **&** P<0.01 *Vs* Vehicle group (3w PI, EMSCs group: F=1.78, P<0.0001; PMECs group: F=0.22, P<0.0001. 8w PI, EMSCs group: F=2.99, P<0.0001; PMECs group: F=1.39, P<0.0001). CNTF: *P<0.05*Vs* Normal group (3w Vehicle group: F=4.32, P<0.0001; EMSCs group: F=0.24, P=0.02; 8w PI, Vehicle group: F=1.01, P<0.0001; EMSCs group: F=0.47, P=0.0003) **&** P<0.01 *Vs* Vehicle group (3w PI, EMSCs group: F=0.15, P=0.002; PMECs group: F=0.04, P=0.0009. 8w PI, EMSCs group: F=0.46, P<0.0001; PMECs group: F=0.48, P<0.0001). # P<0.05 *Vs* EMSCs group (3w PI, PMSCs group: F=0.64, P=0.04; 8w PI, PMSCs group: F=0.97, P=0.005). (J-O): In vehicle-treated EAE rats, the expression of active caspase-3 is markedly increased and the expression of NF-200 was evidently down regulated. This phenomenon could be reversed by EMSCs and

PMSCs transplantation. Caspase-3: *****P<0.05*Vs* Normal group (3w Vehicle group: F=2.38, P<0.0001; EMSCs group: F=0.29, P<0.0001. PMECs group: F=0.31, P<0.0001; 8w PI, Vehicle group: F=0.01, P=0.0004; EMSCs group: F=0.02, P=0.009. PMECs group: F=0.01, P=0.001) **&** P<0.01 *Vs* Vehicle group (3w PI, EMSCs group: F=0.12, P<0.0001; PMECs group: F=0.13, P<0.0001. 8w PI, EMSCs group: F=1.49, P=0.001; PMECs group: F=0.53, P=0.0006) NF=200: *****P<0.01*Vs* Normal group (3w Vehicle group: F=0.44, P<0.0001; EMSCs group: F=0.11,P=0.0009; PMECs group: F=0.08,P=0.001. 8w PI, Vehicle group: F=0.48,P<0.0001) **&** P<0.01 *Vs* Vehicle group (3w PI, EMSCs group: F=0.25,P<0.0001; PMECs group: F=0.18, P<0.0001. 8w PI, EMSCs group: F=3.85,P<0.0001; PMECs group: F=1.26, P<0.0001). (P-R): GFAP expression visibly increased at both week 3 and week 8 following EAE induction, but decreased following EMSCs and PMSCs treatments as shown by Western blotting. *****P<0.01*Vs* Normal group (3w Vehicle group: F=0.3, P<0.0001; 8w PI, Vehicle group: F=3.6, P<0.0001) **&** P<0.01 *Vs* Vehicle group (3w PI, EMSCs group: F=1.93, P<0.0001; PMECs group: F=2.56, P<0.0001. 8w PI, EMSCs group: F=0.07, P<0.0001; PMECs group: F=0.2, P<0.0001). Data are represented as mean ± SD. n=5, degrees of freedom=n-1.

Figure S9. EMSCs and PMSCs treatments reduce neuronal loss. Visible neuronal loss is observed in the brain cortex (B) and spinal cord (F) in CNS of the vehicle-treated EAE rats at 8 weeks post-injection, when compared with healthy animals (A, E). Nevertheless, in EMSCs and PMSCs-treated EAE rats, more neurons are present in the anterior horn of the spinal cord (G-H) and in the motor cortices (C-D). Nissl staining, scale bar = 100 mm. (I-K): The

transplanted EMSCs and PMECs obviously infiltrated into spinal cord parenchymal (I) and formed MSCs masses inside the brain parenchymal tissue (J, K). (L): Number of surviving neural cells calculated in the different groups at 8 weeks post-injection following Nissl staining (each group is presented as a percentage of the healthy control). Data are represented as mean ± SD. N=5, degrees of freedom=4. *P<0.01 *Vs* Normal group (In SP, Vehicle group: F=0.15, P<0.0001; EMSCs group: F=0.14, P=0.003; PMECs group: F=0.06, P=0.0018. In BC, Vehicle group: F=1.18, P<0.0001; EMSCs group: F=1.02, P<0.0001; PMECs group: F=0.6, P<0.0001) **&** P<0.01 *Vs* Vehicle group (In SP, EMSCs group: F=0.94, P<0.0001; PMECs group: F=0.37, P<0.0001. In BC, EMSCs group: F=0.86, P<0.0001; PMECs group: F=0.51, P=0.0001.) # P<0.01 *Vs* EMSCs group (In SP, PMSCs group: F=0.37, P=0.013).

Supplemental figures

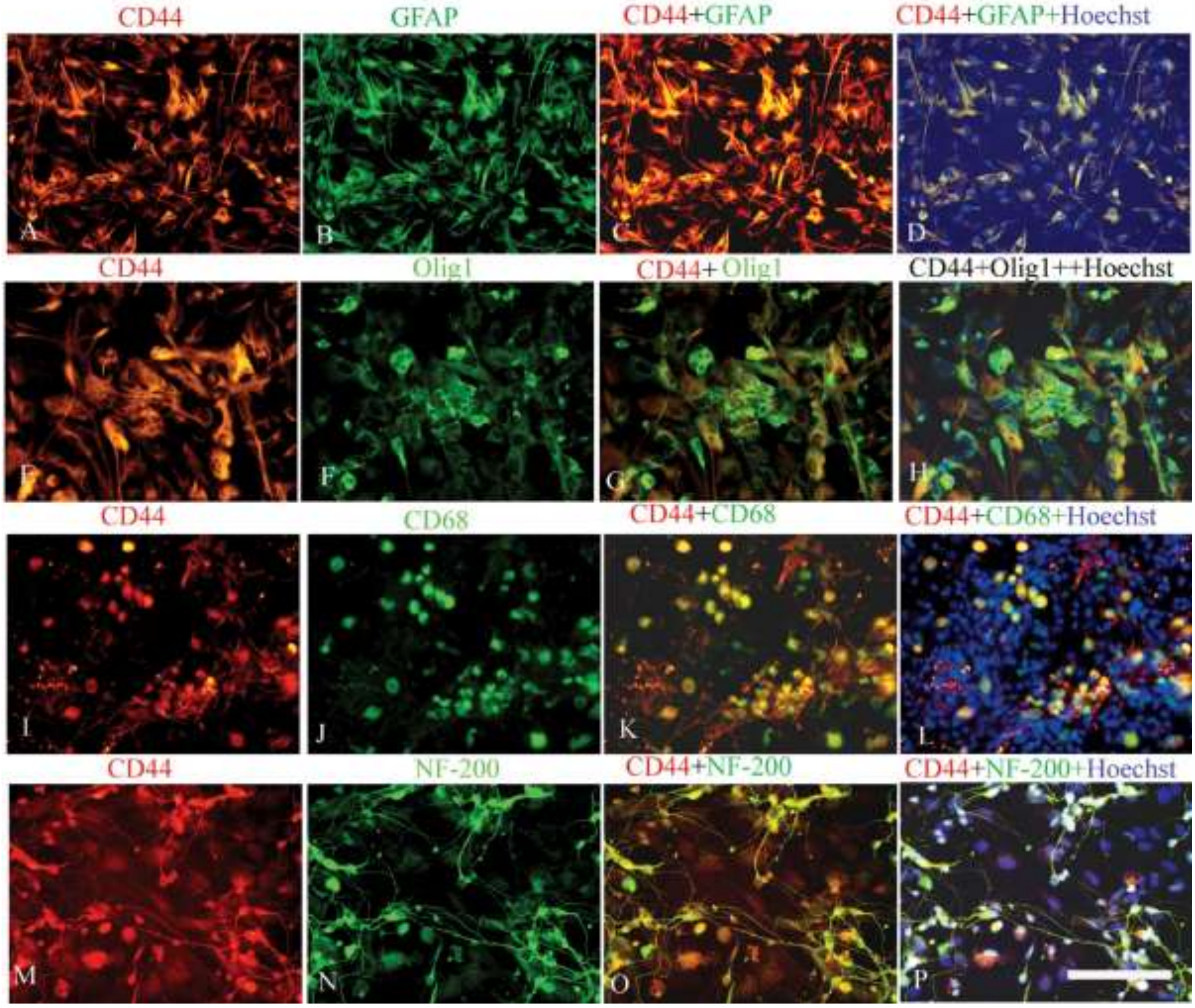

Figure S1

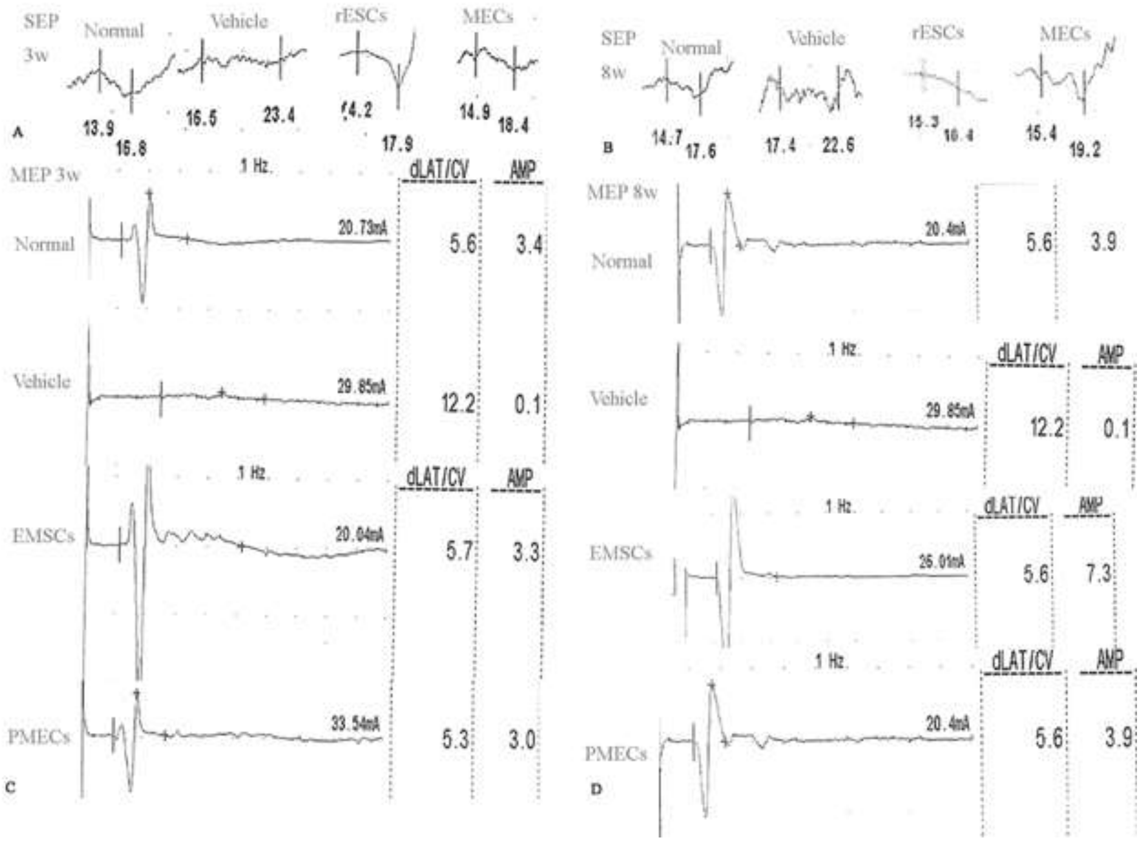
SEP
3w
Normal
Vehicle
rESCs
MECs
A
13.9
16.8
16.5
23.4
17.9
14.9
18.4
SEP
8w
Normal
Vehicle
rESCs
MECs
B
14.7
17.6
17.4
22.6
15.4
19.2
MEP 3w
1 Hz
dLAT/CV
AMP
Normal
20.73mA
5.6
3.4
Vehicle
29.85mA
12.2
0.1
EMSCs
20.04mA
5.7
3.3
PMECs
33.54mA
5.3
3.0
C
MEP 8w
Normal
20.4mA
5.6
3.9
Vehicle
29.85mA
12.2
0.1
EMSCs
26.01mA
5.6
7.3
PMECs
20.4mA
5.6
3.9
D

Figure S2

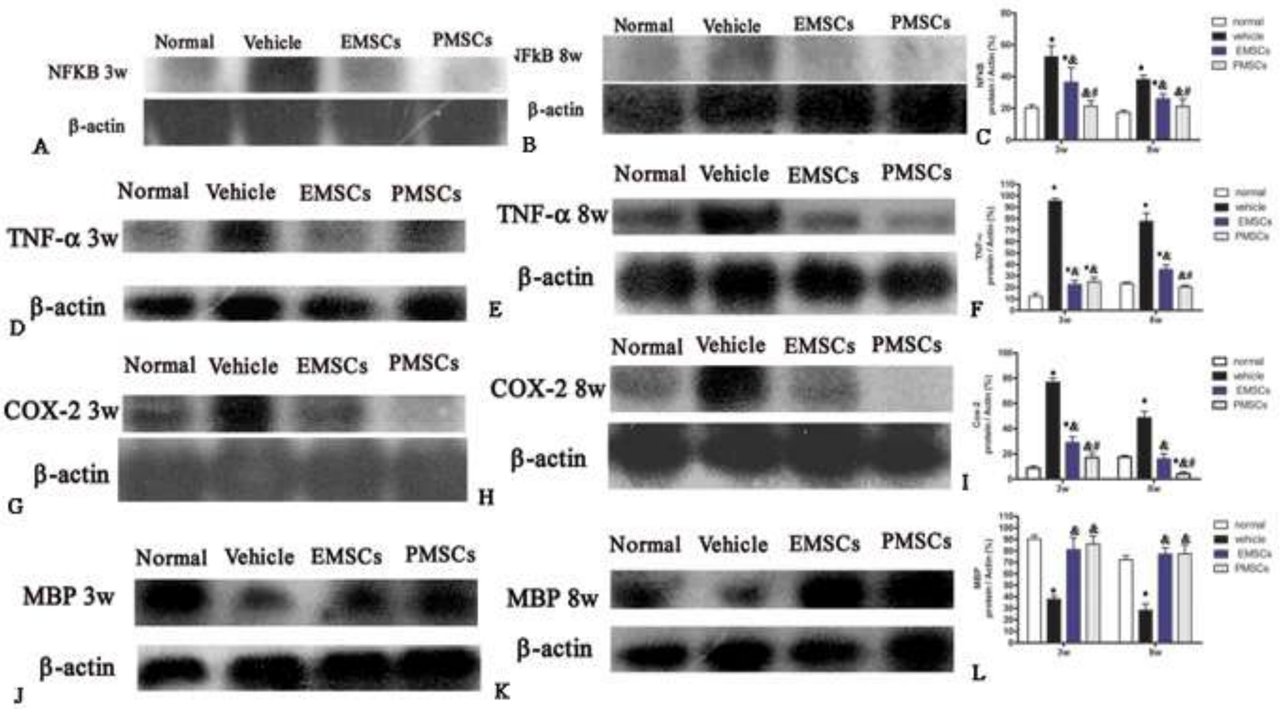
Normal
Vehicle
EMSCs
PMSCs
NFKB 3w
β-actin
A
NFkB 8w
B
C
TNF-α 3w
D
TNF-α 8w
E
F
COX-2 3w
G
COX-2 8w
H
I
MBP 3w
J
MBP 8w
K
L
normal
vehicle
EMSCs
PMSCs
3w
8w

Figure S3

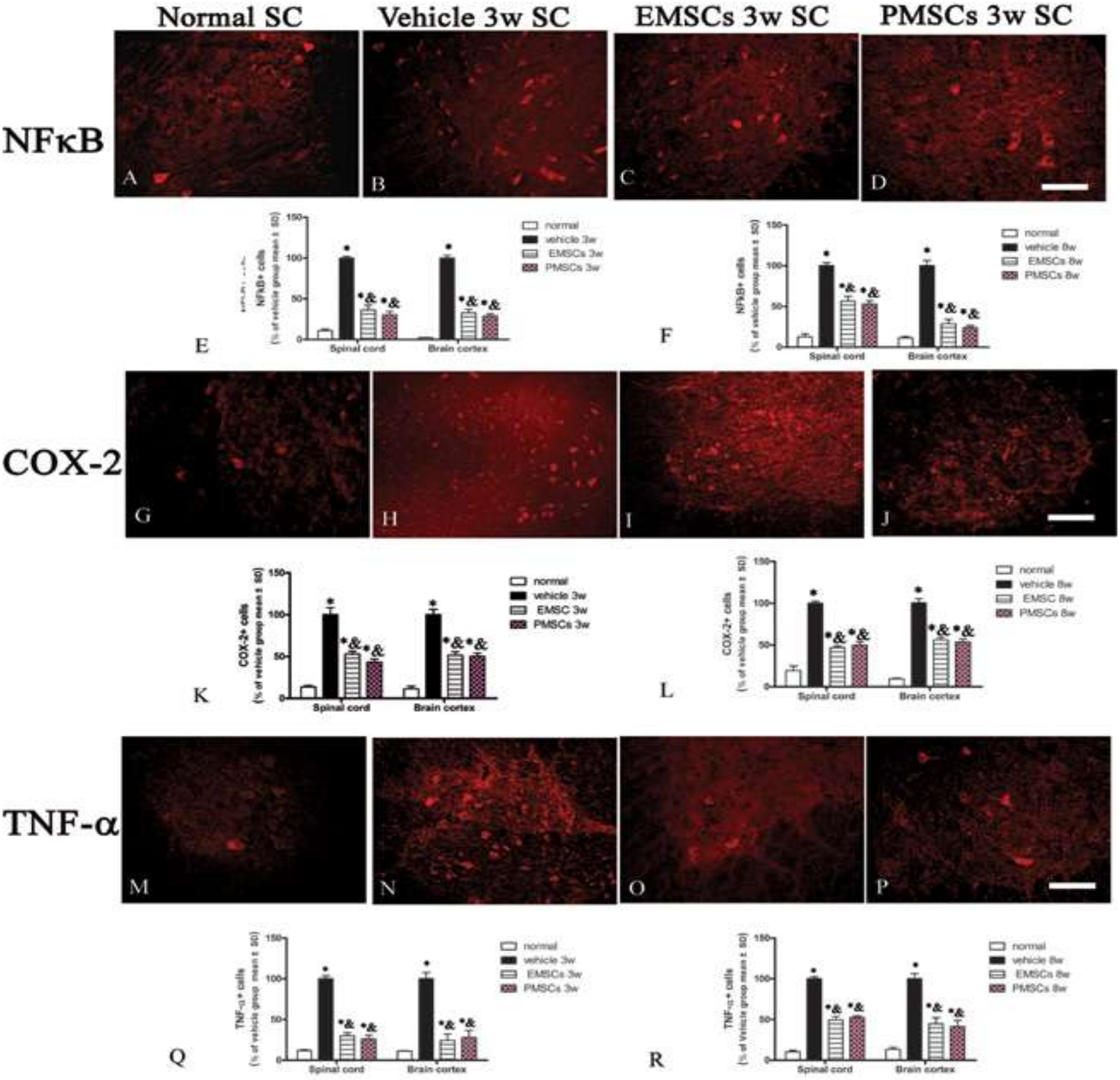
Normal SC
Vehicle 3w SC
EMSCs 3w SC
PMSCs 3w SC
NFκB
A
B
C
D
E
F
COX-2
G
H
I
J
K
L
TNF-α
M
N
O
P
Q
R
Spinal cord
Brain cortex
normal
vehicle 3w
EMSCs 3w
PMSCs 3w
vehicle 8w
EMSCs 8w
PMSCs 8w
EMSC 3w
EMSC 8w

Figure S4

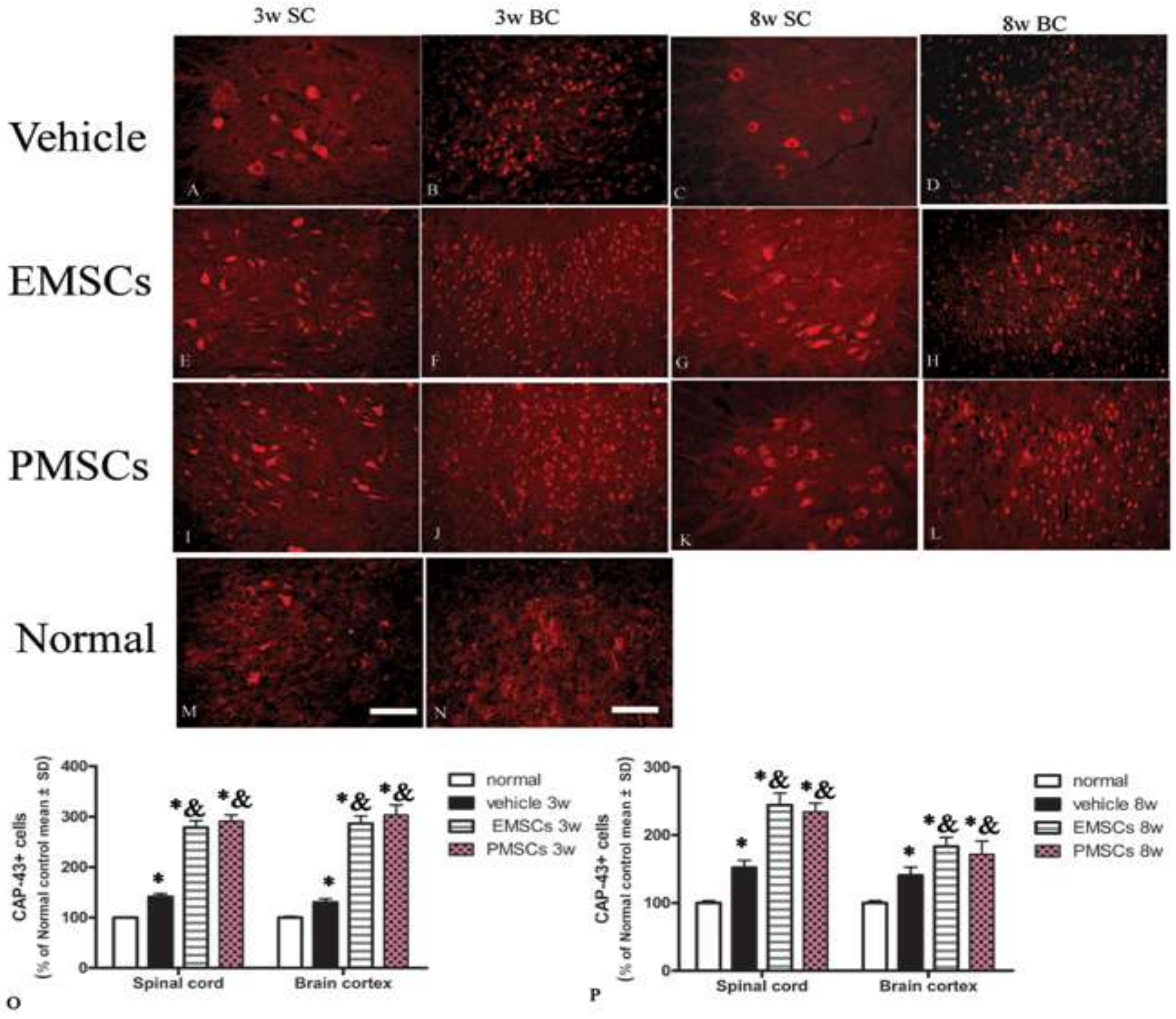

3w SC
3w BC
8w SC
8w BC
Vehicle
EMSCs
PMSCs
Normal
A
B
C
D
E
F
G
H
I
J
K
L
M
N
CAP-43+ cells
(% of Normal control mean ± SD)
400
300
200
100
0
Spinal cord
Brain cortex
normal
vehicle 3w
EMSCs 3w
PMSCs 3w
O
300
200
100
0
normal
vehicle 8w
EMSCs 8w
PMSCs 8w
P

Figure S5

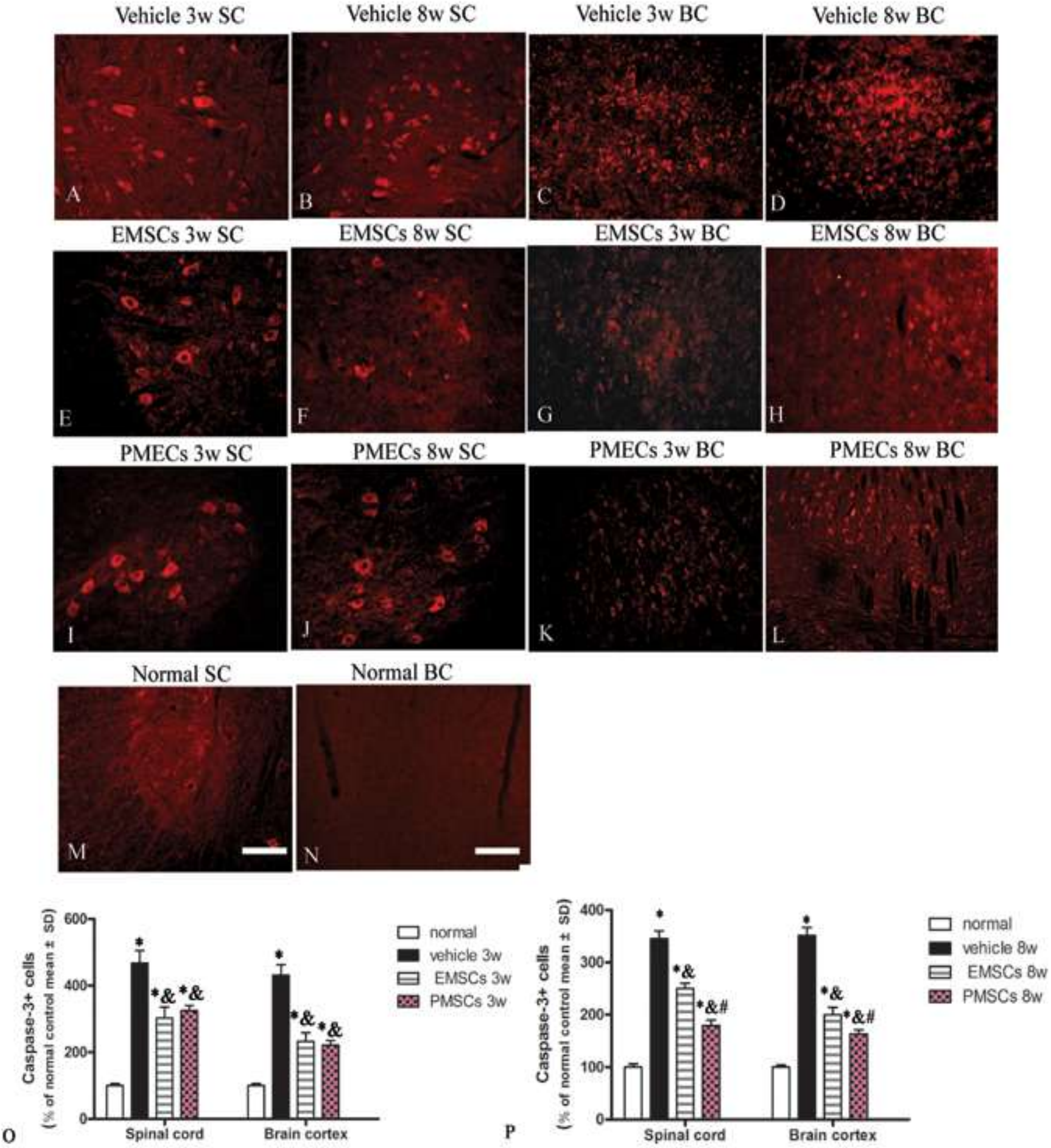

Vehicle 3w SC
Vehicle 8w SC
Vehicle 3w BC
Vehicle 8w BC
A
B
C
D
EMSCs 3w SC
EMSCs 8w SC
EMSCs 3w BC
EMSCs 8w BC
E
F
G
H
PMECs 3w SC
PMECs 8w SC
PMECs 3w BC
PMECs 8w BC
I
J
K
L
Normal SC
Normal BC
M
N
Caspase-3+ cells
(% of normal control mean ± SD)
600
400
200
0
Spinal cord
Brain cortex
normal
vehicle 3w
EMSCs 3w
PMSCs 3w
O
Caspase-3+ cells
(% of normal control mean ± SD)
400
300
200
100
0
Spinal cord
Brain cortex
normal
vehicle 8w
EMSCs 8w
PMSCs 8w
P

Figure S6

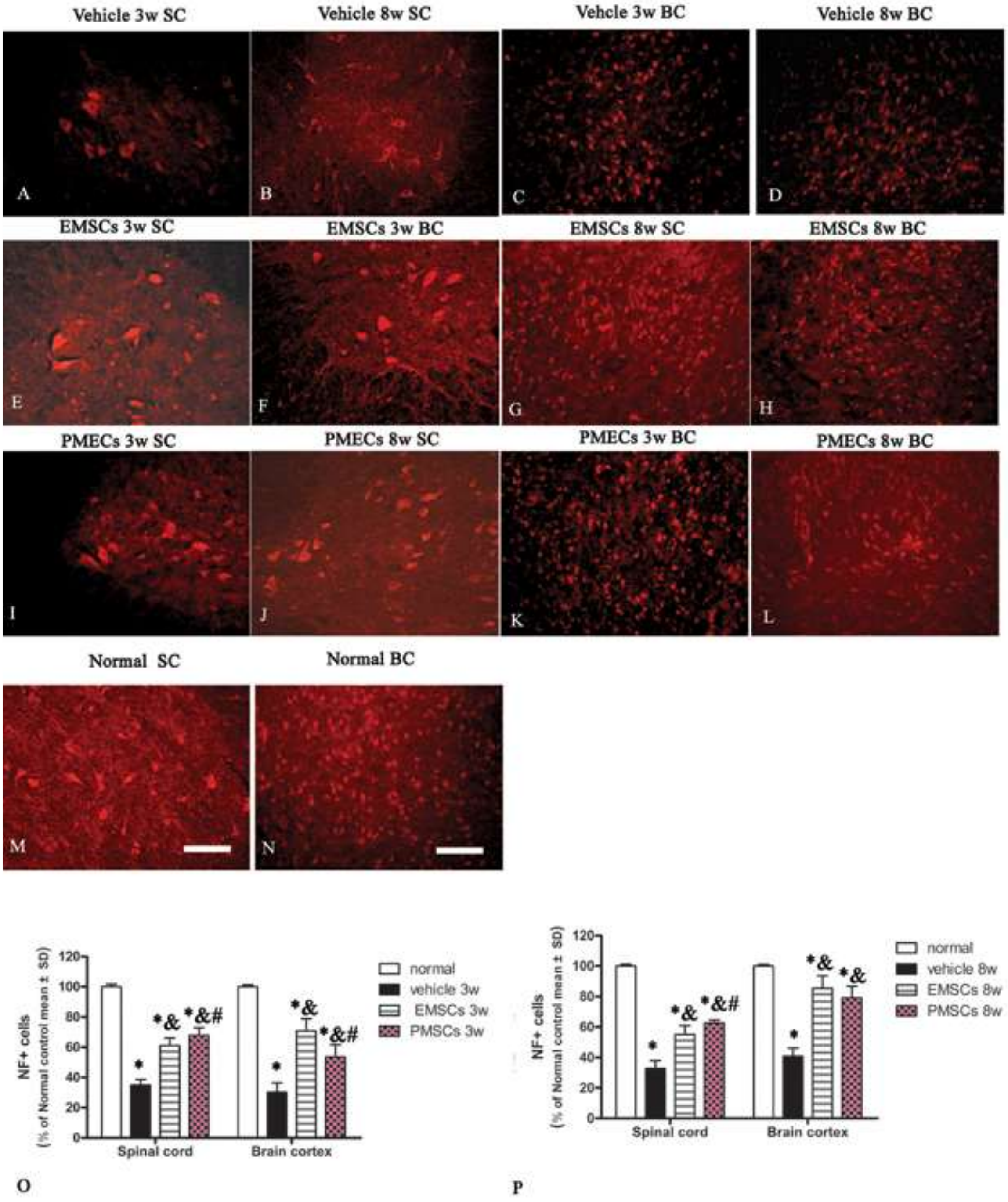
Vehicle 3w SC
Vehicle 8w SC
Vehcle 3w BC
Vehicle 8w BC
A
B
C
D
EMSCs 3w SC
EMSCs 3w BC
EMSCs 8w SC
EMSCs 8w BC
E
F
G
H
PMECs 3w SC
PMECs 8w SC
PMECs 3w BC
PMECs 8w BC
I
J
K
L
Normal SC
Normal BC
M
N
NF+ cells
(% of Normal control mean ± SD)
Spinal cord
Brain cortex
normal
vehicle 3w
EMSCs 3w
PMSCs 3w
vehicle 8w
EMSCs 8w
PMSCs 8w
O
P

Figure S7

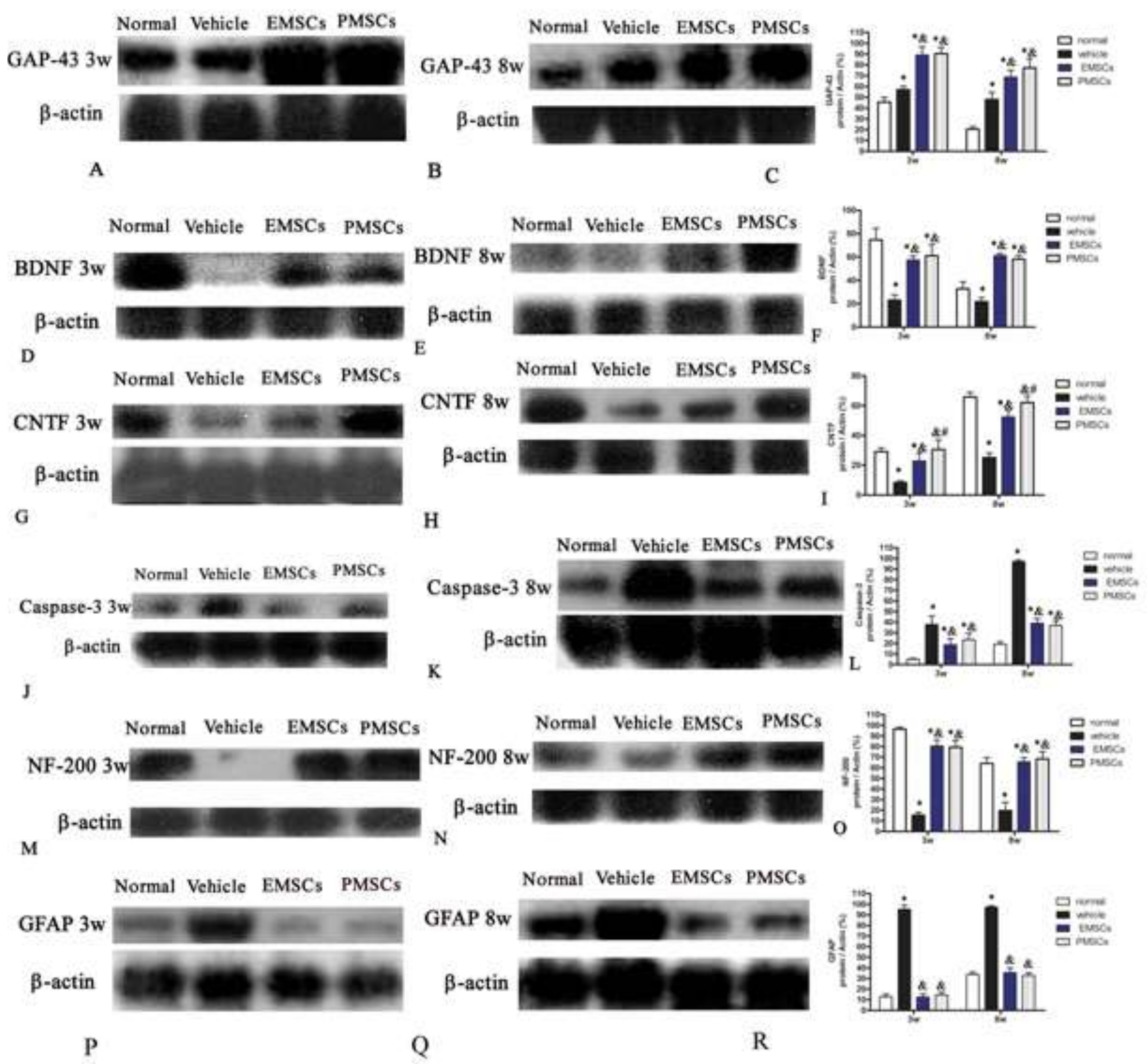

Normal
Vehicle
EMSCs
PMSCs
GAP-43 3w
GAP-43 8w
BDNF 3w
BDNF 8w
CNTF 3w
CNTF 8w
Caspase-3 3w
Caspase-3 8w
NF-200 3w
NF-200 8w
GFAP 3w
GFAP 8w
β-actin
A
B
C
D
E
F
G
H
I
J
K
L
M
N
O
P
Q
R

Figure S8

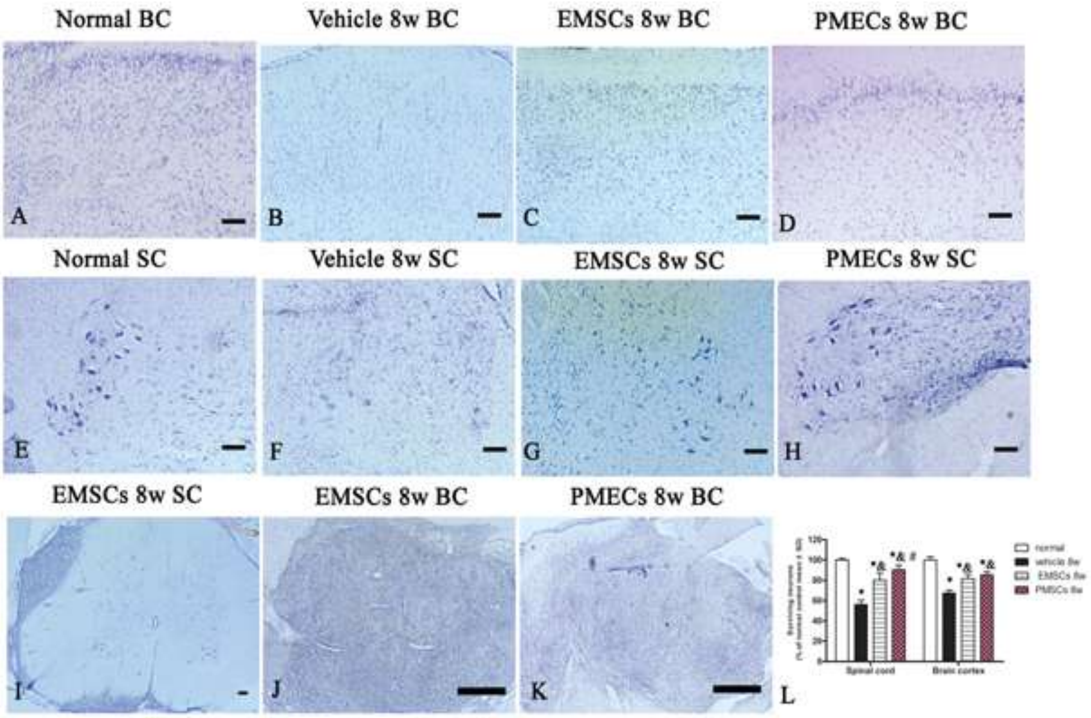
Normal BC
Vehicle 8w BC
EMSCs 8w BC
PMECs 8w BC
A
B
C
D
Normal SC
Vehicle 8w SC
EMSCs 8w SC
PMECs 8w SC
E
F
G
H
EMSCs 8w SC
EMSCs 8w BC
PMECs 8w BC
I
J
K
L
Spinal cord
Brain cortex
normal

Figure S9
